# Simultaneous analysis of large-scale RNAi screens for pathogen entry

**DOI:** 10.1186/1471-2164-15-1162

**Published:** 2014-12-22

**Authors:** Pauli Rämö, Anna Drewek, Cécile Arrieumerlou, Niko Beerenwinkel, Houchaima Ben-Tekaya, Bettina Cardel, Alain Casanova, Raquel Conde-Alvarez, Pascale Cossart, Gábor Csúcs, Simone Eicher, Mario Emmenlauer, Urs Greber, Wolf-Dietrich Hardt, Ari Helenius, Christoph Kasper, Andreas Kaufmann, Saskia Kreibich, Andreas Kühbacher, Peter Kunszt, Shyan Huey Low, Jason Mercer, Daria Mudrak, Simone Muntwiler, Lucas Pelkmans, Javier Pizarro-Cerdá, Michael Podvinec, Eva Pujadas, Bernd Rinn, Vincent Rouilly, Fabian Schmich, Juliane Siebourg-Polster, Berend Snijder, Michael Stebler, Gabriel Studer, Ewa Szczurek, Matthias Truttmann, Christian von Mering, Andreas Vonderheit, Artur Yakimovich, Peter Bühlmann, Christoph Dehio

**Affiliations:** Focal Area Infection Biology, Biozentrum, University of Basel, Klingelberstrasse 70, CH-4056 Basel, Switzerland; Seminar for Statistics, ETH Zurich, Zurich, Switzerland; Institute of Molecular Life Sciences, University of Zurich, Zurich, Switzerland; Department of Biosystems Science and Engineering, ETH Zurich, Zurich, Switzerland; Swiss Institute of Bioinformatics, Basel, Switzerland; Unité des Interactions Bactéries Cellules; INSERM, U604; INRA, USC2020, Institut Pasteur, Paris, France; Institute for Tropical Health and Departamento de Microbiología y Parasitología, Universidad de Navarra, Pamplona, Spain; Institute of Biochemistry, ETH Zurich, Zurich, Switzerland; Department of Biology, Institute of Microbiology, ETH Zurich, Zurich, Switzerland; Institute of Molecular Biology, Mainz, Germany; Light Microscopy and Screening Center, ETH Zurich, Zurich, Switzerland; Research IT, Biozentrum, University of Basel, Basel, Switzerland; Institut Cochin, INSERM U1016, CNRS 8104, Université Paris Descartes, Paris, France; SyBIT, SystemsX.ch, Zurich, Switzerland

**Keywords:** High-throughput high-content RNAi screening, Pathogen entry, Linear mixed model, Hit detection

## Abstract

**Background:**

Large-scale RNAi screening has become an important technology for identifying genes involved in biological processes of interest. However, the quality of large-scale RNAi screening is often deteriorated by off-targets effects. In order to find statistically significant effector genes for pathogen entry, we systematically analyzed entry pathways in human host cells for eight pathogens using image-based kinome-wide siRNA screens with siRNAs from three vendors. We propose a Parallel Mixed Model (PMM) approach that simultaneously analyzes several non-identical screens performed with the same RNAi libraries.

**Results:**

We show that PMM gains statistical power for hit detection due to parallel screening. PMM allows incorporating siRNA weights that can be assigned according to available information on RNAi quality. Moreover, PMM is able to estimate a sharedness score that can be used to focus follow-up efforts on generic or specific gene regulators. By fitting a PMM model to our data, we found several novel hit genes for most of the pathogens studied.

**Conclusions:**

Our results show parallel RNAi screening can improve the results of individual screens. This is currently particularly interesting when large-scale parallel datasets are becoming more and more publicly available. Our comprehensive siRNA dataset provides a public, freely available resource for further statistical and biological analyses in the high-content, high-throughput siRNA screening field.

**Electronic supplementary material:**

The online version of this article (doi:10.1186/1471-2164-15-1162) contains supplementary material, which is available to authorized users.

## Background

Large-scale RNAi screening is a widely used technology to knock-down expressions of genes and study their protein function in a biological process of interest [1–5]. In several published studies in the field of infection biology, cells perturbed with siRNAs were exposed to pathogens and differences in phenotypic outcomes were measured in order to identify the genes involved in successful infection or to develop functional models of host signaling and trafficking pathways [6–9].

RNAi libraries are mostly sold in formats containing enough material for numerous large-scale screens. Therefore, several large-scale siRNA screens are typically performed using the same libraries within a unit such as a university or company in order to optimize material costs and to simplify plate handling. However, parallel screens are typically performed and analyzed separately without common protocols or analysis pipelines. Therefore, comparing results between the screens is challenging. Co-operative efforts, such as assays using common key parameters for imaging and data analyses, could enable to produce more comparable results and gain parallel information for each individual screen. In the field of RNAi screening, there has been progress in relation to the standardization of data publication formats, in particular in the context of the “Minimum Information About an RNAi Experiment” (MIARE, http://miare.sourceforge.net) and GenomeRNAi [10] efforts. However, the provided metadata information and data analysis approaches are often diverse so that data comparison between the screens from different laboratories is very difficult.

Poor reproducibility rates of large-scale RNAi screens are a common concern. They are mostly caused by strong off-target effects from particular siRNAs [11–16]. Strategies have been proposed to alleviate the confounding effects of RNAi screens, including experimental [17, 18] and statistical approaches [9, 19–22]. In this study, we aim to use the parallel screening structure in order to gain statistical power for hit selection in large-scale RNAi screens. We generated high-content siRNA datasets that are uniquely comprehensive in terms of the siRNA libraries and various pathogens used. We employed highly unified protocols for parallel screens and common data analysis pipelines to allow a direct comparison between the readouts of different pathogen screens. In addition to obtain a list of hits for individual pathogens, our aim was to discover shared mechanisms between pathogens. To this purpose, we propose a new statistical method – the Parallel Mixed Model (PMM). Our approach simultaneously takes into account the knock-down effects of several non-identical screens performed in parallel with the same RNAi libraries. Additionally, the PMM provides a local False Discovery Rate (FDR) for every gene, resulting in a probability estimate that a gene is a false positive. We will show that the model improves statistical power thanks to parallel screening and that it yields stable hits, novel to the studied pathogens, without compromising the detection of unique hits for any given single screen.

## Results and discussion

### High-content siRNA screening

**Figure 1:**
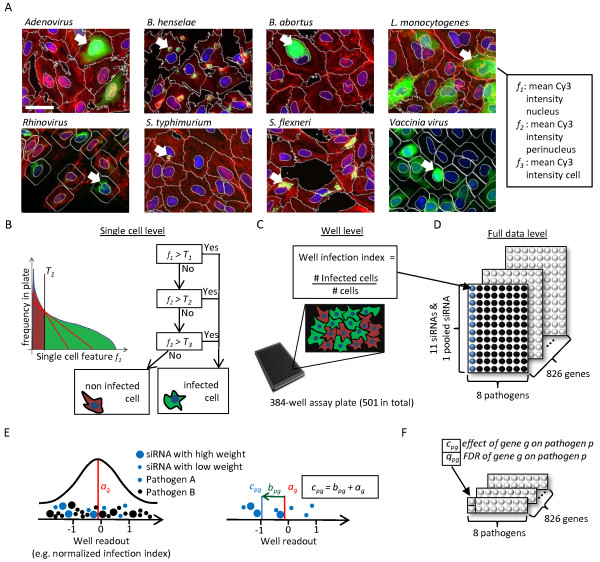
**Overview of InfectX high-content datasets, image analysis, and Parallel Mixed Model (PMM). (A)** The figure shows example images of the different pathogens after siRNA transfection and the infection phase. The arrows indicate typical infectious phenotypes for each pathogen. The list shows an example of three single cell features that we extracted to identify infected cells for *L. monocytogenes*. The scale bar has a length of 50 μm. **(B)** For each selected feature, we defined the optimal threshold that separated best between uninfected and infected cells via histograms. We used the thresholds in the Decision Tree Infection Scoring (DTIS) algorithm to classify between infected (green) and non-infected cells (red). We optimized this procedure for each pathogen separately. **(C)** For each well in a 384-well assay plate, we calculated the infection index by dividing the number of infected cells (green) by the total number of cells (green and red). **(D)** The figure shows a schematic representation of the input data for the statistical analysis. Each point represents the average infection index over all its replicate wells (wells with the same siRNA set targeting the same gene and pathogen). **(E)** The Parallel Mixed Model (PMM) algorithm fits via a normal distribution for an overall effect *a*_*g*_ to all data of gene *g*. The second plot shows the correction of the overall effect *a*_*g*_ within every pathogen by an estimate *b*_*pg*_ in order to obtain to an pathogen and gene specific effect *c*_*pg*_. The different sizes of the data points refer to weights *w*_*s*_ which can be incorporated in the PMM to depict the quality of the siRNA. **(F)** The figure shows a schematic representation of the final output of PMM. The model estimates gene effects *c*_*pg*_ for each gene and pathogen and provides corresponding local False Discovery Rates *q*_*pg*_.

Our InfectX consortium, consisting of eleven research groups, generated kinome-wide siRNA screens for five bacterial pathogens (*Bartonella henselae*, *Brucella abortus*, *Listeria monocytogenes*, *Salmonella typhimurium*, and *Shigella flexneri*) and three viruses (*Adenovirus, Rhinovirus, and Vaccinia virus*) and systematically analyzed the biological pathways leading to successful infection in human host cells (Figure 1). This choice of bacterial and viral pathogens covered a wide variety of mechanism to invade host cells. *B. henselae*, for example, invades host cells by invasome structures [23], the pathogens *S. typhimurium* and *S. flexneri* use the trigger mechanism, while *L. monocytogenes* uses the zipper mechanism [24]. *Adenovirus* and *Rhinoviru*s enter by a dynamin and clathrin dependent pathway [25] and *Vaccinia virus* by macropinocytosis [26].

We conducted the screens in a highly parallel manner under one common protocol for all eight pathogens. We carried out all screens in the same HeLa ATCC-CCL-2 cell line and with the same reagent batches of shared providers. The set of 826 targeted genes comprised almost the whole kinome, plus selected kinome-associated genes, and we targeted each gene by a total of eleven independent siRNAs coming from three manufactures: Ambion (Silencer Select) with 3 siRNAs per gene, Qiagen (Human Kinase siRNA Set V4.1) with 4 siRNAs per gene and Dharmacon (Human ON-TARGETpIus) with 4 siRNAs per gene. Additionally, we performed screens where we targeted each kinase with a pool of the four Dharmacon siRNAs (Human ON-TARGETpIus SMARTpooI). However, not all of the 826 genes have a full set of 11 siRNAs and 1 siRNA pool available. Depending on the pathogen and library, we independently repeated the screens one to six times as replicates (see Additional file 1: Table S1). To obtain an optimal dynamic range of infectivity, we chose the pathogen dose and entry time to be pathogen specific (see Additional file 1: Table S2). We fixed and stained the cells using DAPI or Hoechst to detect nuclei, fluorescent labeled phalloidin to detect actin filaments and the cell body, and a pathogen specific marker to detect infected cells. In a final step, we imaged the screens using microscopes of the same brand. Thus, we only permitted deviations from the common protocols when the infection assay required it.

We separately optimized image analysis for each pathogen and established for each pathogen a list of image features that described the phenotypes of infected cells. For example, for *S. flexneri*, we chose as one feature the RFP intensity of the extracted bacteria objects and for *L. monocytogenes* the mean Cy3 intensity of the cell (see Figure 1A and Additional file 1). In the next step, we classified the cells in each well as infected or uninfected with a Decision Tree Infection Scoring (DTIS) algorithm (see Additional file 1) and obtained a rate of infection per well (infection index) (Figures 1B–C). Besides assay-specific readouts the image analysis also provided several assay-independent readouts (e.g. cell number). We alleviated possible batch effects, dependencies to the population context, and further experimental confounders by data normalization (see Additional file 1) [27–32]. We performed all analyses presented in this paper with the normalized infection index readout, unless otherwise stated.

### Data reproducibility

**Figure 2:**
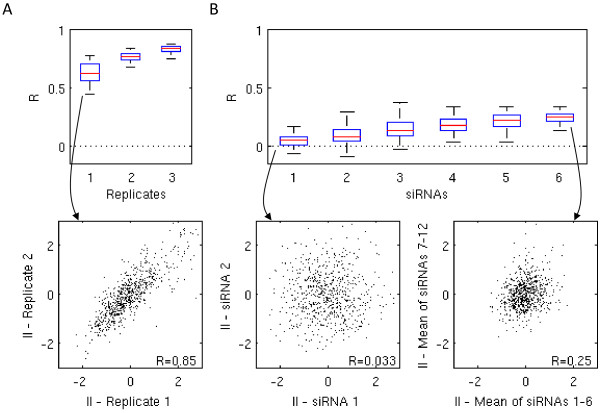
**Using more siRNAs adds power and yields reproducible results. (A)** The three boxplots show Pearson correlation coefficients *R* between screens performed using the same siRNA set. The numbers 1 to 3 correspond to the total number of replicate screens that we averaged and compared to another distinct set of replicate screens, averaged over the same number. We resampled the replicate screens up to 500 times. The scatter plot shows an example for the correlation of infection indices from the duplicate of *Adenovirus* Dharmacon pooled screen. **(B)** The set of six boxplots shows the Pearson correlation coefficients of the averaged readouts from 1 to 6 siRNA sets. The scatter plots depict the correlation of infection indices for *Adenovirus*, the first between two different single siRNAs and the second between each an average over six siRNAs.

Our data confirmed the reported [20] low reproducibility rates of siRNA data originating from different siRNAs targeting the same genes. The normalized infection indices of two different siRNA sets targeting the same genes showed a Pearson correlation coefficient *R* between 0 and 0.2 depending on the screens (Figure 2B). Adding independent siRNAs to the screen yielded an increase in the correlation coefficients, but the correlation still stayed at an unsatisfactory level, even with six separate siRNAs targeting each gene (*R* was between 0.1–0.4 in averaged and separate sets of six independent siRNAs). In contrast, replicate screens (screens performed using the same protocols and siRNA set, but performed at a different time) were reproducible (*R* was between 0.5–0.9) (Figure 2A). For practical reasons, assuming a similar assay quality as ours, performing screens in duplicates seems sufficient since having more replicates does not improve the data to a great extent (Figure 2A). On the other hand, performing screens at least in duplicates is necessary for quality control and performing only single screens is therefore not recommendable. The cell number readouts (see Additional file 1: Figure S4) showed qualitatively similar results for data reproducibility. In summary, the main error source in our siRNA screening was the bias caused by varying specificity of siRNAs and not by technical variability of the screens.

### Parallel Mixed Model (PMM)

Assuming that the sources of variability between different siRNAs targeting the same gene are statistically independent, we can benefit from the fact that the true signal is enhanced by using more siRNAs targeting the individual genes [17] (Figure 2B). In order to increase the statistical power of individual siRNA screens, we performed screens with 11 siRNAs (and one pool of siRNAs) targeting each gene. Moreover, when using the parallel structure in the data and combining data points from all pathogen screens together, we reached 8×12 = 96 data points for every gene (averaging over the replicate screens). We propose the Parallel Mixed Model (PMM) as a suitable approach to model the distribution of the siRNA readouts using all data together, including all available siRNAs and pathogen screens.

PMM is composed of a linear mixed model and an assessment of the local False Discovery Rate (FDR) (Figure 1E–F). The linear mixed model is an extension of the ordinary linear model by random effects [33]. In particular, random effects are not determined by fixed coefficients, but by Gaussian distributions. Therefore, we can incorporate the variation among the siRNAs in form of random effects and estimate all effects for different pathogens simultaneously. To be more precise, the linear mixed model consists of a fixed effect *μ*_*p*_ for pathogen *p* and two random effects *a*_*g*_ for gene *g* and *b*_*pg*_ as a correction term for gene *g* within pathogen *p*:


where *y*_*pgs*_ denotes the readout (for example the normalized infection index of a well) of pathogen *p* and gene *g* knocked-down with siRNA *s* and *ϵ*_*pgs*_ denotes the unobserved error term. We fitted the linear mixed model by using the “lmer” function from the “lme4” R-package [34]. The sum of two random effects *a*_*g*_ and *b*_*pg*_ describes the total effect of the siRNAs within pathogen *p*. We define the estimated effect *c*_*pg*_ for gene *g* within pathogen *p* as


A positive estimated *c*_*pg*_ effect means that the infection level was enhanced if the corresponding gene *g* is knocked down. A negative effect means that the infection level was reduced. To distinguish hit genes, PMM provides as second step an estimate *q*_*pg*_ of the local False Discovery Rate (FDR). We computed the local False Discovery Rate using the approach presented in [59] and the “locfdr” function in the R-package of the same name [35]. We assigned the local False Discovery Rate to every gene and interpreted it as the probability describing how likely the corresponding gene is a false discovery (see Methods for more details). The PMM method is published as “PMM” R-package on the InfectX data-access page.

**Figure 3:**
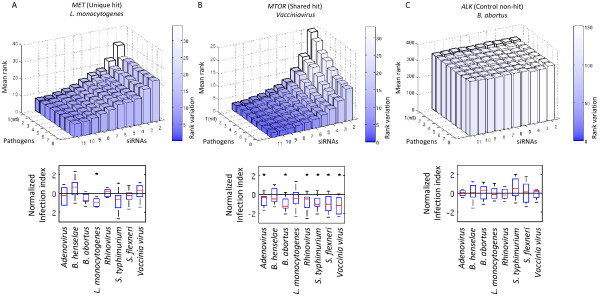
**Parallel screens add power to find more shared hits. (A)** We varied the number of used siRNAs and pathogens and calculated the rank of *MET* for *L. monocytogenes* in the ordered list of hit genes. We used PMM (and MTT for the case of one pathogen) over 1000 random resampling rounds with replacement. The color corresponds to the variation of the observed ranks. The boxplot shows that *MET* is a unique strong hit among the studied pathogens. The star indicates the boxplots that are significantly different from 0 (one sample t-test p < 0.05). **(B)** The figure shows the same experiment as in **(A)**, but now with *MTOR* for *Vaccianiavirus*. The boxplot shows that *MTOR* is a shared significant hit for several pathogens. **(C)** The figure shows the same experiment as in **(A)** but with non-hit *ALK* for *B. abortus* for control.

As a first verification for the increase in power by simultaneously using the parallel screening structure, we resampled datasets, each consisting of a fixed number of siRNAs and pathogens, and fitted the PMM, respectively the Moderated T-Test (MTT) [36] for the case of one pathogen (see Methods for details). We evaluated the mean and variation (i.e. stability) of the ranks in the ordered lists of genes based on their estimated *c*_*pg*_ values over 1000 resampling runs for *MET* (a known effector gene for *L. monocytogenes*[37]), *MTOR* (a role of *MTOR* in the infection pathways of several pathogens has already been established [6, 15, 38]) and a non-hit *ALK* as control (Figure 3). The results showed, in particular in the case of *MTOR*, that the rank and its stability improved by simultaneously using more siRNAs and pathogens. In the case of *MET* the use of parallel screens did not cause an increase in statistical power, since *MET* was a hit for *L. monocytogenes* only. However, for *MET* there was no reduction of statistical power either. These examples already indicated that the parallel screening structure and PMM can be used to more reliably discover expected effector genes even in the case where only a fraction of effector genes is shared between the screens.

### Analysis of siRNA libraries

**Figure 4:**
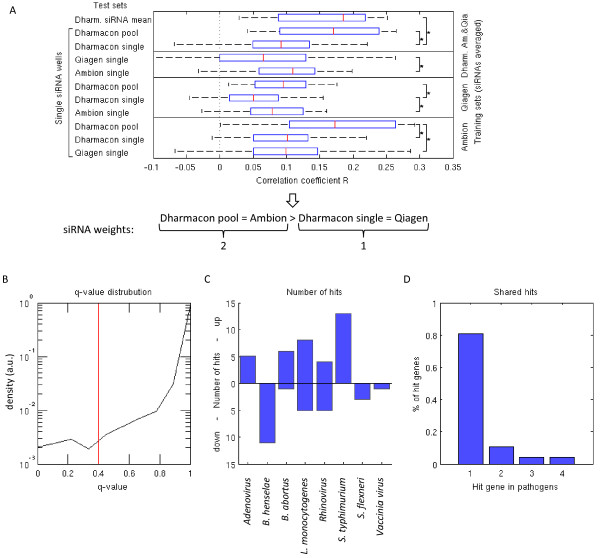
**Statistics on used siRNA libraries and hits. (A)** We weighted siRNAs based on their library quality. Each vertical compartment in the plot corresponds to a training set of siRNAs. We averaged data in the training set from the siRNAs of the specific manufacturers. Each boxplot corresponds to a test set of single siRNAs from different manufacturers (except “Dharm. siRNA mean” which is the average of 4 Dharmacon unpooled siRNAs). Y-axis refers to Pearson correlation coefficients *R* between the training and test sets. A star corresponds to significant differences in the correlation coefficients (Mann–Whitney-U-test p < 0.05) between pairs of manufacturers. We used all screens, infection index, and cell number well readouts in the analysis. We used the results of this analysis to assign siRNA weights to siRNAs from different library manufacturers as shown below the plot. **(B)** The histogram shows obtained FDR q-values from all screens using the infection index readouts. The red line shows the FDR-threshold of 0.4. **(C)** The bar shows number of up and down hits for different pathogens. **(D)** The bar plot shows the number of hit genes that were shared between pathogens.

PMM allows the assignment of weights to each siRNA (see Methods). With weighting, we can assign more power to siRNAs that are estimated to have little off-target effects and strong knock-down efficiencies. Within this study, we weighted siRNAs according to the reproducibility in terms of correlation of their corresponding library to other libraries (Figure 4A). There are several potential other ways how weights could be determined. However, we did not follow them further within the context of this paper.

We cross-validated different libraries to each other by fixing one library manufacturer (training set) at a time (Figure 4A). We averaged phenotypic readouts from siRNAs targeting the same gene in the training set in order to obtain reference gene readouts. In this analysis we used both infection index and cell number readouts. We then compared single siRNA readouts of the remaining two library manufactures (test set) to the reference gene readouts. The Pearson correlation coefficients of the test sets enable to quantify which of the two test manufacturers produces more reproducible results. By repeating the procedure for all manufacturers as the training set we could order the manufacturers in terms of their reproducibility performance. Our results based on phenotypic readouts showed that the pooled Dharmacon library performed the best. The pooled library was followed by the unpooled libraries of Ambion, Dharmacon, and Qiagen in this order. However, there were no statistically significant differences (Wilcoxon rank-sum test p < 0.05) between Dharmacon pooled and Ambion single, and Dharmacon single and Qiagen single siRNA data reproducibility. In addition, the data showed that the averaged single siRNAs of Dharmacon performed at most as good as the single pooled siRNA consisting of the same siRNAs. This indicated that for most screening purposes, it is more practical to use the pooled library instead of several unpooled libraries. This result of better performance of pooled libraries compared to averaged single siRNA libraries is in contradiction with what has been reported in [19]. However, good quality single siRNA libraries (such as Ambion Silencer Select) performed nearly as well as pooled libraries of less good single siRNAs (in our case Dharmacon SMARTpool). Following the results of the library analysis, we assigned a higher weight to Dharmacon Pooled and Ambion libraries (weight 2) than to the unpooled libraries Dharmacon and Qiagen (weight 1). PMM benefitted from the assigned library weights. The residual standard error of the linear mixed model reduced from 0.87 to 0.83.

### Sharedness of detected significant genes

**Figure 5:**
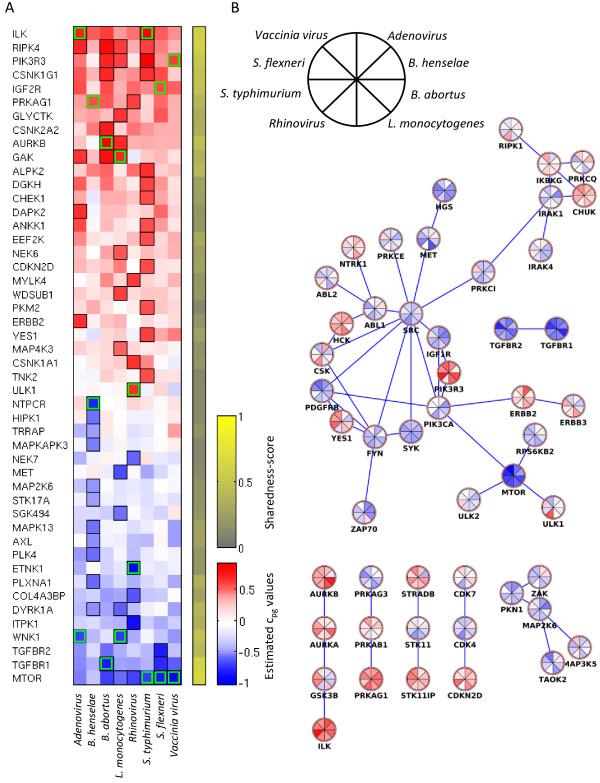
**Summary of screening hits for all pathogens. (A)** The heat map shows all genes which were significant (FDR < 0.4) at least for one pathogen. We ordered the genes by their averaged c-values over all pathogens. The colors correspond to the estimated c-values. The black outlines indicate significant hits (FDR < 0.4) and the green outlines high-light the strongest down and up hits for each pathogen. The rightmost column shows the sharedness scores for each gene. **(B)** The network shows the hit genes (FDR < 0.4 for at least one pathogen) and their direct neighbors that had connections between kinases in STRING database (version 9.0). The edges are functional interactions in the STRING database with edge threshold 850. We removed genes that were not connected to any other gene from the network. Each node consists of a colored pie chart, in which each piece corresponds to a pathogen.



By fitting PMM to our data, we found a left tailed local False Discovery Rate distribution, ending with a set of 50 different genes that reached the threshold of 0.4 (Figure 4B, Figure 5A). We selected threshold 0.4 as a reasonable hit threshold for this study since the difference was small compared to the set of hits with the commonly used threshold 0.2 and 40% false-positive rate was still acceptable in biological follow-up studies for us. The number of up and down hits varied between the pathogens (Figure 4C). Using FDR threshold 0.4, 80% of hits were unique and 20% of hits were shared between two or more studied pathogens (Figure 4D). This provided a rough estimate that about 20% of genes gained statistical power from the parallel analysis using the PMM with our data. To quantify the hits according to their level of being shared between screens independently from the FDR-threshold, we developed the following “sharedness score” *s*_*g*_:

Here *P* is the total number of pathogens (8 in our case). The sharedness score is a combination of two quantities. The first part defines the shift away from 1 and the second part describes how many pathogens support this shift (proportion of *q*_*pg*_ < 1). The score returns a value between 0 and 1 for each gene. Score 0 indicates that a gene is not shared among the pathogens and score 1 indicates that the gene is significant among all pathogens (Figure 5A). Since the sharedness score takes only the strength of a gene and not the directionality into account, a gene can be also highly shared if it inhibits in one pathogen and enhances the infection by another pathogen. Therefore, a gene shared between pathogens should be interpreted as being involved in the entry of these pathogens.

### Result comparison to existing hit ranking methods

In order to validate the PMM approach and its results we compared it to other existing hit ranking methods and performed different kind of statistical tests. As reference methods we selected the Moderated T-Test (MTT) [36] and Redundant SiRNA Activity (RSA) [39] which are commonly used in high-throughput RNAi screening. We could not apply other widely used hit ranking methods, such as Strictly Standardized Mean Difference (SSMD) [40] or percent inhibition [29] since many of our pathogen screens did not have effective and reliable positive and negative control wells.

**Figure 6:**
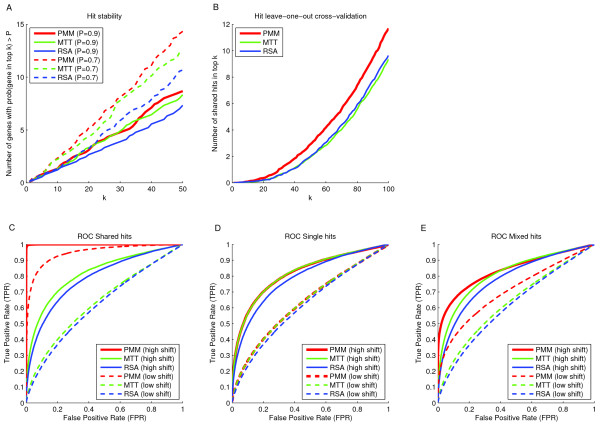
**Performance statistics of hit ranking methods. (A)** The figure shows stability curves using the three different methods (PMM, MTT and RSA). The y-axis denotes the number of genes that were found with probability higher than 0.7 (dashed lines) and 0.9 (solid lines) in the top k (x-axis) of the list of ranked genes. The curves show the average over all eight pathogens. **(B)** The figure shows hit overlaps of cross-validated siRNA sets between the set of 10 unpooled siRNA libraries and the remaining siRNA library using the three tested gene ranking methods as a function of hit threshold *k*. The curves show the average over all eight pathogens. **(C)** The figure shows ROC-curves for PMM, MTT and RSA applied on simulated data containing only hits that were shared between all pathogens. The dashed and solid lines indicate whether the shifts were generated by a low or high shift away from zero. The PMM method outperformed the reference hit detection method. **(D)** The figure shows ROC-curves for PMM, MTT and RSA applied on simulated data containing only unique hits for all pathogens. PMM and Moderated T-Test performed equally well. **(E)** The figure shows ROC-curves for simulated data with a mixed hit structure of both unique and shared hits. The PMM method outperformed the reference hit detection method.

As a first test, we analyzed the stability of the obtained gene rankings with respect to the estimated *c*_*pg*_ values [30, 41]. We resampled with replacement 1000 datasets (12 siRNAs randomly selected with replacement for each gene) and calculated the number of genes that appear with high probability (prob > 0.9 and prob > 0.7) in the top of the ordered lists of genes based on their estimated *c*_*pg*_ values (see Methods for details). This measure of stability showed similar results for PMM and the reference methods MTT and RSA (Figure 6A).

To mimic primary and validation screening setup and to study hit reproducibilities of the gene ranking methods we performed a leave-one-out cross-validation experiment. We used the siRNAs of unpooled libraries (11 in total) and left one siRNA set at a time away. We ran PMM, MTT, and RSA on the data sets consisting of 10 individual siRNAs and compared the resulting gene ranking to the ranked gene list of the remaining siRNA set. The averaged hit overlaps over all pathogens as a function of hit threshold *k* are illustrated in Figure 6B. PMM performed the best indicating that the hits found by PMM are more reproducible by an independent siRNA screen than the hits found by the other methods.

In order to further estimate the hit-calling performance for different methods we performed data simulation with a-priori known hit structure. Data simulation was required since reliable ground truth hits are not generally available for the real biological systems. We simulated data by generating 1000 Gaussian distributed screens for each pathogen with four siRNAs. We selected four siRNAs since it makes up a realistic screening approach. We incorporated hits in each simulated screen by randomly selecting 10% of the genes and shifting them away from zero. We distinguished between three types of simulated data. In the first case the hits were different for each pathogen (unique hits only) and in the second case all hits were shared between the pathogens. The third case is probably the most realistic scenario containing both unique and shared hits to a varying degree (see Methods). We then applied PMM, MTT, and RSA to the simulated data and evaluated them by Receiver Operating Characteristic (ROC) -curves (with false positive versus true positive rates plotted for each FDR- threshold; Figure 6C–E). The results showed that PMM performed the best especially in the case of shared hits. For the case of unique hits PMM and MTT exhibited about the same performance while RSA performed the worst. As expected, with a higher shift of the hit genes the ROC curves got better for all methods.

**Figure 7:**
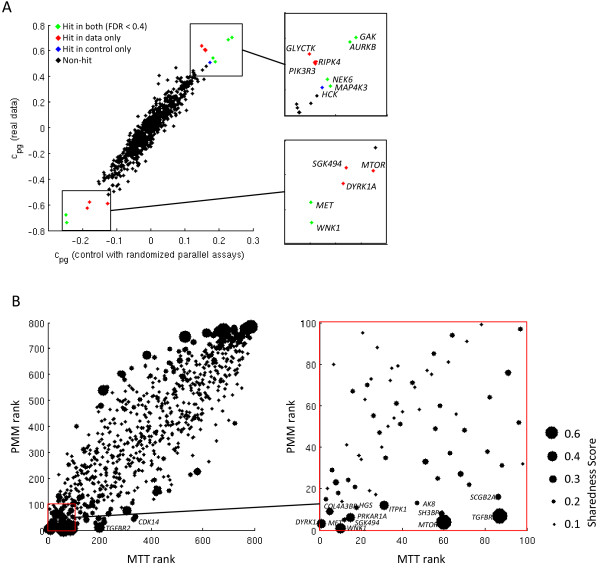
**Summary of differences of PMM top hits compared to other hit scoring methods. (A)** Y-axis shows the PMM gene ranking for *L. monocytogenes.* X-axis is the same, but we randomized the other 7 parallel assays. The colors correspond to hit genes (FDR < 0.4) in different cases. Parallelism yielded only a slight effect on the ranking, but added genes to the list of significant hit genes. **(B)** The scatter plot shows PMM hit ranking (y-axis) compared to the MTT hit ranking (x-axis) for *L. monocytogenes*. The dot size corresponds to the sharedness score of each gene. Some genes with high sharedness scores gained statistical power.

We also studied how simultaneous modeling affects the ranking of genes in individual screens using PMM. We performed a test where we selected a pathogen and created two datasets. The first dataset was the full data without any changes and the second dataset had the original data for the selected pathogen and randomized data for the 7 other pathogens. We then compared the gene rankings obtained by PMM performed using both datasets for the selected pathogen. The results for *L. monocytogenes* are illustrated in Figure 7A (see Additional file 1: Figure S6 for all the other pathogens). The correlation graph shows that the addition of parallel screens had only a mild effect on the overall gene ranking. However, when considering the number of significant genes (FDR < 0.4), PMM mainly added genes to the list of significant genes (7 novel significant genes for *L. monocytogenes*) and only few genes (1 for *L. monocytogenes*) were dropped off the list. In general, we concluded that using parallelism added novel significant genes while losing almost none. Moreover, the few lost hit genes had high FDR values, just slightly below the selected threshold FDR < 0.4.

In the next step we analyzed the differences between the resulting gene rankings of the tested methods. Differences in gene rankings between PMM and other hit ranking methods were not very strong (see Figure 7B for MTT compared to PMM for *L. monocytogenes* and Additional file 1: Figure S7 for all other cases). Genes that had a high sharedness score and had an effect on the screen of interest (in particular *MTOR* and *TGFBR1/2* for *L. monocytogenes*) gained statistical power from the simultaneous analysis and were pushed up in the gene ranking. On the other hand, we observed that PMM detected several genes with low sharedness scores, indicating that unique hits were not neglected.

In order to evaluate the biological relevance of observed hits, we calculated pathway enrichment scores separately for each pathogen by the Gene Set Enrichment Analysis (GSEA) algorithm [42] using as input the results from the three hit ranking algorithms PMM, MTT, and RSA (see Additional file 1). We selected all pathways that were significant (GSEA pathway enrichment FDR score < 0.2) for at least one pathogen and method pair. We used the ranking of infection indices as the input for GSEA and focused on hits that reduce infection levels. By assuming that most pathways in the used database are biologically valid, we would expect that better hit detection methods give a higher number of enriched pathways than less powerful hit detection methods. However, we only screened kinases and the applicable pathways are limited to those that are highly enriched in phosphorylation events and it may be that some pathogens do not show strong enrichments within this set of pathways. Moreover, differences in pathway enrichments between methods may have occurred because they treated missing values differently. Therefore, the enrichment results should be evaluated with caution. Additional file 1: Figure S9 illustrates the observed significant pathways. The number of enriched (GSEA FDR < 0.2) pathways for each method was an indication that PMM detected biologically more relevant hit genes than the other methods.

### Biological inquiry on detected significant genes

The performed screens yield several interesting hits of which most are novel to the corresponding pathogen (Figure 5A, see Additional file 1: Figure S10 for cell number hits). Many of the strongest hits, including *MTOR*, *TGFBR1/2* for negative hits and *ILK* for positive hits, were shared between most of the studied pathogens. This was also illustrated by the sharedness scores of detected hit genes. Many of the strongest shared hits were related to *SRC, MTOR*, or *CDK* related pathways. Although *SRC* and *CDK4* were not part of the hit lists (*q*_*pg*_ < 0.4) for any of the pathogens, they exhibited consistent semi-strong effect for most pathogens. A network analysis of hit genes showed that several of the shared hits can be described as “network hubs” that are involved in many cellular processes and highly connected to other genes (including *MTOR* and *SRC*) (Figure 5B) [43]. *MTOR* is a mammalian target of rapamycin, serine/threonine protein kinase that regulates cell growth, cell proliferation, cell motility, cell survival, protein synthesis, and transcription. The involvement of *MTOR* in *Adenovirus*, *Poliovirus*, *Enterovirus71*, *Coxackievirus, Vaccinia virus* and other pathogens has already been established [6, 9, 15]. Our data also reproduced the established role of *MTOR* during *S. typhimurium* infections, since *S. typhimurium* depends on a reactivation of *MTOR* during its course of infection in order to escape autophagy [38]. Interestingly, *TGFBR1* and *TGFBR2* came up both as strong hits for many pathogens. *TGFBR1* and *TGFBR2* proteins must heterodimerize to form a functional TGF-beta receptor at the plasma membrane. Their similar strong infection reducing knock-down phenotypes, seen in most independent pathogen screens, indicated the validity of these hits and suggested a broad, yet poorly understood, function of this membrane protein for various pathogens. In particular, there are suggestions [44] that the *TGFB* pathway might be important for *B. abortus* infection since in chronic brucellosis patients there is increased *TGF beta* production and this could aid infection by depressing lymphocyte functions. In addition, our study confirmed the role of *DYRK* family members (in particular *DYRK1A*) as they have been identified to be general regulators for several viruses in Snijder, Sacher et al. [9].

Despite the overall similarity of infection patterns between pathogens, most pathogens also contained hits that were specific for the pathogen (for example *MET* for *L. monocytogenes*, *NTPCR* for *B. henselae*, and *ETNK1* and *ULK1* for *Rhinovirus*). Some of the hit genes have previously been found to be effectors, for example *MET* for *L. monocytogenes. L. monocytogenes* enters host cells by triggering signaling cascades activated through interaction of bacterial *internalin A* (*InlA*) or *InlB* with the adherens junction protein *E-cadherin* or the hepatocyte growth factor receptor *MET*[37] respectively. Since E-cadherin is not expressed in HeLa cells, which were used for our siRNA screens, the *INLB / MET* pathway is the only route of entry in this cellular system. In fact, *MET*[45] was one of the strongest hits for *L. monocytogenes*. The exact roles of most hit genes of all pathogens are largely unknown, but several hit genes create interesting hypotheses for follow-up. For example, it was proposed based on microRNA analysis of infected macrophages, that *AMPK* might be a target gene that promotes intracellular survival during *B. abortus* infection [46]. *PIK3R3* (p55-gamma; Phosphatidylinositol 3-Kinase 55 KD a Regulatory Subunit Gamma) a semi-strong hit for several pathogens in our data was identified as a hit in an RNAi screen of *drosophila* S2 cells, in agreement with the importance of *PI3K* during *B. abortus* infection [47]. *PIK3CA* probably plays a role also in *B. henselae* infection through actin modulation. *PIK3CA* levels influence *RHOA* and *RAC1*, which are involved in actin dynamics [48]. Furthermore, *PIK3CA* is involved in *PIP3* production, which is a signaling molecule and has recently been shown to be related to the formation of dynamic F-actin-related structures [49]. *ULK1* (*unc-51* like autophagy activating kinase 1) plays an important role in autophagy as well as *Hepatitis C virus* infection. Therefore, *ULK1* has a possible link to *Rhinovirus* induced autophagy. *COL4A3BP* is possibly linked to *Rhinovirus* entry through ceramide-enriched membrane platforms [50] since *COL4A3BP* specifically phosphorylates the N-terminal region of the non-collagenous domain of the alpha 3 chain of type IV collagen, known as the Goodpasture antigen, also involved in ceramide intracellular transport (from ER to Golgi).

## Conclusions

We produced a uniquely wide high-content siRNA dataset, in terms of used siRNA libraries (11 single siRNAs and one pool) and eight different pathogens. Our highly unified protocols and common image analysis as well as similar data analysis pipelines enabled a direct comparison between the phenotypic readouts of the different pathogen screens. The unified structures of the datasets also aided discovering shared mechanisms between the studied pathogens.

Using our novel statistical approach PMM we detected several interesting and new hits from our kinome-wide pathogen screens. The hits will require further follow-up work in order to understand the exact biological mechanisms of the genes. In addition, we discovered shared effector genes between the studied pathogens including *MTOR*, *TGFBR1* and *TGFBR2* that were strong hits for almost all studied pathogens. In particular, the obtained sharedness scores indicated whether a hit gene has a very specific function for a single pathogen or a more generic cellular function that is shared between many pathogens and thus gave us the first indications of the gene’s roles. Pharmaceutically oriented follow-up studies could take advantage of this concept. For example, if we were interested in general regulators we could focus on genes with high sharedness scores. On the other hand, regulators that have a very specific effect and a low sharedness score could probably have fewer side effects.

We showed that the reliability of hit scoring in individual RNAi screens improved by using PMM that takes advantages of the parallelism in RNAi screening. PMM can, in principle, be applied to any kind of parallel RNAi screens almost independently of the underlying biology or field of application as long as the readouts of the screens are measured on the same scale. We can often obtain this by applying Z-Scoring or similar normalization methods to the well readouts. The difference to other approaches aiming at the comparison of independent parallel RNAi screens is that PMM takes simultaneously all screening data into account. For example, for the comparison of insect and human data in [51] the hit lists were derived by separate statistics on each screen. By taking all data into the analysis the statistical power can be increased. Based on our results, we expect that the more similar the parallel screens are in the sense of biological focus or protocols, the more statistical power can be gained from the simultaneous analysis. Even a slight overlap between the underlying biological pathways of the parallel screens can improve the hit detection in individual screens without compromising the detection of unique hits for any individual screens. Provided that the large-scale RNAi screening community reaches standardized data publication and sharing standards through projects such as MIARE and GenomeRNAi, the PMM approach could be expanded to include the vast number of different RNAi screens performed in different laboratories worldwide that used the same siRNA libraries. In principle and as a vision, this opens up great opportunities for simultaneous statistical approaches such as PMM. Every new screen could potentially gain statistical power by using the public resources. In addition, PMM can potentially be used to gain power for secondary validation screens. Such validation screens are typically performed with several independent siRNAs targeting the same gene under various conditions and PMM would be directly applicable. A beneficial feature of PMM is the possibility to assign weights to the siRNAs. The weights can incorporate a-priori information about the performance of individual siRNAs and their phenotypical readout. This concept of weighting can be expanded over what we presented in this paper. In particular, statistical and bioinformatics analyses on seed sequence induced off-target effects could potentially be used as basis for weights. Naturally any additional high-throughput data, such as proteomics analyses on cells under siRNA perturbations, or genomic analyses on specific cell lines, could be used to assign realistic siRNA weights to improve hit scoring.

We aimed to take a step forward in determining minimal requirements for image-based RNAi screening data publication. All the raw images, library metadata, single cell measurements, and well measurements are publicly available through our openBIS based publication portal. In addition, we provide easy-to-access data aggregates in standardized tabular formats with all the necessary metadata information. Our uniquely wide datasets provide a large resource for infection biologists, image analysts, and statisticians for future research.

## Methods

### Wet-lab protocols

#### Cell culturing conditions

HeLa CCL-2 (ATCC) cells were maintained at 37°C and 5% CO_2_ in Dulbecco Modified Eagle Medium (DMEM, Invitrogen) supplemented with 10% inactivated FCS (Invitrogen).

### siRNA reverse transfection

RNA interference directed against human kinases and kinase-associated genes (826 genes in total) was achieved using commercially available siRNA libraries. All experiments were conducted in a 384-well plate format. In addition to screening plates, control plates were included in each screen. All plates contained general siRNA controls for transfection efficiency and toxicity (e.g. Kif11), as well as, control siRNAs for infection effects of each pathogen assayed. However, for most of the pathogens in this study, reliable and well established positive control siRNAs (reducing or enhancing infection levels) were not available prior to screening. In addition, negative controls such as *MOCK* (no siRNA) and *SCRAMBLED* (non-targeting siRNA) were added to every plate.

In each experiment, 25 μl of RNAiMAX/DMEM (0.1 μl/24.9 μl) mixture was added to each well of the screening plates containing 1.6 pmol siRNA diluted in 5 μl RNase-free ddH_2_O. Screening plates were thereafter incubated at room temperature (RT) for 1 h. Following incubation, a pathogen assay-specific number of HeLa CCL-2 cells (see Additional file 1: Table S1) were added per well in a volume of 50 μl DMEM/16% FCS, resulting in a final FCS concentration of 10% (*Adenovirus* screens contained 6.7% final FCS). Plates were incubated at 37°C and 5% CO_2_ for 72 h prior to infection.

### Fixation and staining

After infection cells were fixed using paraformaldehyde (PFA). Cells were stained for DNA, F-actin and infection specific markers. Screening plates were sealed prior to imaging.

### *Adenovirus*-specific protocol

All liquid handling stages of infection, fixation, and immunofluorescence staining were performed on the automated pipetting system Well Mate (Thermo Scientific Matrix) and washer Hydrospeed (Tecan). For infection screens recombinant Ad2_ΔE3B-eGFP (short *Adenovirus*) was utilized as described before [52, 53]. *Adenovirus* was added to cells at a multiplicity of infection (moi) of 0.1 in 10 μl of an infection media/FBS (DMEM supplemented with L-glutamine, 10% FBS, 1% Pen/Strep, Invitrogen). Screening plates were incubated at 37°C for 16 h, and cells were fixed by adding 21 μl of 16% PFA directly to the cells in culture media for 45 min at RT or long-term storage at 4°C. Cells were washed 2 times with PBS/25 mM NH_4_Cl, permeabilized with 25 μl 0.1% Triton X-100 (Pharmaciebiothek). After 2 washes with PBS the samples were incubated at RT for 1 h with 25 μl staining solution (PBS) containing DAPI (1 μg/ml, Sigma-Aldrich) and DY-647-phalloidin (1 U/ml, Dyomics),washed 2 times with PBS and stored until imaging in 50 μl PBS/NaN_3_.

### *Bartonella henselae*-specific protocol

Bacterial strain SEB0109: *Bartonella henselae* ATCC49882^T^ Δ*bepG* containing plasmid pCD353 [54] for IPTG-inducible expression of GFP. Culturing conditions: bacteria were grown on Columbia base agar (CBA) plates supplemented with 5% defibrinated sheep blood (Oxoid) and 50 μg/ml kanamycin. Bacteria were incubated at 35°C in 5% CO_2_ for 72 h before re-streaking them on fresh CBA and further growth for 48 h. Infection: siRNA-transfected cells were washed once with M199 (Invitrogen)/10% FCS using a plate washer (ELx50-16, BioTek). Cells were infected with *B. henselae* at an MOI of 400 in 50 μl of M199/10% FCS and 0.5 mM IPTG (Applichem) and were incubated at 35°C in 5% CO_2_ for 30 h. Fixation at RT: using a Multidrop 384 (Thermo Scientific) cells were washed with 50 μl of PBS, fixed in 20 μl of 3.7% PFA for 10 min, and washed once more with 50 μl of PBS. Staining on a Biomek liquid handling platform: fixed cells were washed twice with 25 μl of PBS and blocked in PBS/0.2% BSA for 10 min. Extracellular bacteria were labeled with a rabbit serum 2037 against *B. henselae*[23] and a secondary antibody goat anti rabbit Alexa Fluor 647 (Jackson Immuno) in PBS/0.2% BSA. Antibodies were incubated for 30 min each and both incubations were followed by two washings with 25 μl of PBS. Cells were then permeabilized with 20 μl of 0.1% Triton X-100 (Sigma) for 10 min and afterwards washed twice with 25 μl of PBS, followed by the addition of 20 μl of staining solution (PBS containing 1.5 U/ml DY-547-Phalloidin (Dyomics) and 1 ug/ml DAPI (Roche)). After 30 min of incubation in the staining solution, cells were washed twice with 25 μl PBS, followed by a final addition of 50 μl of PBS.

### *Brucella abortus*-specific protocol

*Brucella abortus* 2308 pJC43 (*aphT::GFP*) [55] were grown in tryptic soy broth (TSB) medium containing 50 μg/ml kanamycin for 20 h at 37°C and shaking (100 rpm) to an OD of 0.8-1.1. 50 μl of DMEM/10% containing bacteria was added per well to obtain a final moi of 10000 using a cell plate washer (ELx50-16, BioTek). Plates were then centrifuged at 400 g for 20 min at 4°C to synchronize bacterial entry. After 4 h incubation at 37°C and 5% CO_2_, extracellular bacteria were killed by exchanging the infection medium by 50 μl medium supplemented with 10% FCS and 100 μg/ml gentamicin (Sigma). After a total infection time of 44 h cells were fixed with 3.7% PFA for 20 min at RT with the cell plate washer. Staining was performed using a Biomek liquid handling platform. Cells were washed twice with PBS and permeabilized with 0.1% Triton X (Sigma) for 10 min. Then, cells were washed twice with PBS, followed by addition of 20 μl of staining solution which includes DAPI (1 μg/ml, Roche) and DY-547-phalloidin (1.5 U/ml, Dyomics) in 0.5% BSA in PBS. Cells were incubated with staining solution for 30 min at RT, washed twice with PBS, followed by final addition of 50 μl PBS.

### *Listeria monocytogenes*-specific protocol

After washing an overnight culture of *L. monocytogenes* EGDe.PrfA*GFP three times with PBS, bacteria were diluted in DMEM supplemented with 1% FCS. Cells were infected at a moi of 25 in 30 μl infection medium per well. After centrifugation at 1000 rpm for 5 min and incubation for 1 h at 37°C in 5% CO_2_ to allow the bacteria to enter, extracellular bacteria were killed by exchanging the infection medium by 30 μl DMEM supplemented with 10% FCS and 40 μg/ml gentamicin (Gibco). Both medium exchange steps were carried out with a plate washer (ELx50-16, BioTek). After additional 4 h at 37°C in a 5% CO_2_ atmosphere, cells were fixed for 15 min at RT by adding 30 μl of 8% PFA in PBS to each well using a multidrop 384 device (Thermo Electron Corporation). PFA was removed by four washes with 500 μl PBS per well using the Power Washer 384 (Tecan). Fixed cells were stained for nuclei, actin and bacterially secreted *InlC*. First, cells were incubated for 30 min with 10 μl/well of primary staining solution (0.2% saponin, PBS) containing rabbit derived anti-InlC serum (1:250). After four washes with 40 μl PBS per well cells were stained with 10 μl/well of the secondary staining solution (0.2% saponin, PBS) containing Alexa Fluor-546 coupled anti-rabbit antibody (1:250, Invitrogen), DAPI (0.7 μg/ml, Roche), and DY-647-Phalloidin (2 U/ml, Dyomics). After four washes with 40 μl PBS per well, the cells were kept in 40 μl PBS per well. The staining procedure was carried out with a Tecan freedom evo robot.

### *Rhinovirus*-specific protocol

All liquid handling stages of infection, fixation, and immunofluorescence staining were performed on the automated pipetting system Well Mate (Thermo Scientific Matrix) and washer Hydrospeed (Tecan). For infection assays with human *Rhinovirus* serotype 1a (HRV1a) were carried out as described, except that the anti-VP2 antibody Mab 16/7 was used for staining of the infected cells as described earlier [56–58]. *Rhinovirus* at a moi of 8 was added to cells in 20 μl of an infection media/BSA (DMEM supplemented with GlutaMAX, 30 mM MgCl_2_ and 0.2% BSA, Invitrogen). Screening plates were incubated for 7 h at 37°C, and cells were fixed by adding 33 μl of 16% PFA directly to the culture medium. Fixation was either for 30 min at RT or long term storage at 4°C. Cells were washed twice with PBS/25 mM NH_4_Cl, permeabilized with 50 μl 0.2% Triton X-100 (Sigma-Aldrich) followed by 3 PBS washes and blocking with PBS containing 1% BSA (Fraction V, Sigma-Aldrich). Fixed and permeabilized cells were incubated at RT for 1 h with diluted mabR16-7 antibody (0.45 μg/ml) in PBS/1% BSA. Cells were washed 3 times with PBS and incubated with 25 μl secondary staining solution (PBS/1% BSA) containing Alexa Fluor 488 secondary antibody (1 μg/ml, Invitrogen), DAPI (1 μg/ml, Sigma-Aldrich), and DY-647-phalloidin (0.2 U/ml, Dyomics). Cells were washed twice with PBS after 2 h of incubation in secondary staining solution and stored in 50 μl PBS/NaN_3_.

### *Salmonella typhimurium*-specific protocol

All liquid handing stages of infection, fixation, and immunofluorescence staining were performed on a liquid handling robot (BioTek; EL406). For infection the *S.typhimurium* strain *S.*Tm^SopE_pM975^ was used. This strain is a single effector strain, only expressing SopE out of the main four SPI-1 encoded effectors (SipA, SopB, SopE2 and SopE). Additionally this strain harbors a plasmid (pM975) that expresses GFP under the control of a SPI2 (*ssaG*)-dependent promoter. The bacterial solution was prepared by cultivating a 12 h culture in 0.3 M LB medium containing 50 μg/ml streptomycin and 50 μg/ml ampicillin. Afterwards a 4 h subculture (1:20 diluted from the 12 h culture) was cultivated in 0.3 M LB medium containing 50 μg/ml streptomycin, which reached an OD_600nm_ ≈ 1.0 after the respective 4 h of incubation time. To perform the infection, 16 μl of diluted *S. typhimurium* (moi = 80) were added to the HeLa cells. After 20 min of incubation at 37°C and 5% CO_2_, the *S. typhimurium*-containing media was replaced by 60 μl DMEM/10% FCS containing 50 μg/μl streptomycin and 400 μg/μl gentamicin to kill all remaining extracellular bacteria. After additional 3 h 40 min incubation at 37°C and 5% CO_2,_ cells were fixed by adding 35 μl 4% PFA, 4% sucrose in PBS for 20 min at RT. The fixation solution was removed by adding 60 μl PBS containing 400 μg/ml gentamicin. Cells were permeabilized for 5 min with 40 μl 0.1% Triton X-100 (Sigma-Aldrich). Afterwards 24 μl of staining solution containing DAPI (1:1000, Sigma-Aldrich) and DY-547-phalloidin (1.2 U/ml, Dyomics) was added (prepared in blocking buffer consisting of 4% BSA and 4% Sucrose in PBS). After 1 h of incubation at RT, cells were washed three times with PBS followed by the addition of 60 μl PBS containing 400 μg/ml gentamicin.

### *Shigella flexneri*-specific protocol

*S. flexneri* M90T Δ*virG* pCK100 (PuhpT::dsRed) were harvested in exponential growth phase and coated with 0.005% poly-L-lysine (Sigma-Aldrich). Afterwards, bacteria were washed with PBS and resuspended in assay medium (DMEM, 2 mM L-Glutamine, 10 mM HEPES). 20 μl of bacterial suspension was added to each well with a final moi of 15. Plates were then centrifuged for 1 min at 37°C and incubated at 37°C and 5% CO_2_. After 30 min of infection, 75 μl were aspirated from each well and monensin (Sigma) and gentamicin (Gibco) were added to a final concentration of 66.7 μM and 66.7 μg/ml, respectively. After a total infection time of 3.5 h, cells were fixed in 4% PFA for 10 min. Liquid handling was performed using the Multidrop 384 (Thermo Scientific) for dispension steps and a plate washer (ELx50-16, BioTek) for aspiration steps. For immunofluorescent staining, cells were washed with PBS using the Power Washer 384 (Tecan). Subsequently, cells were incubated with a mouse anti-human IL-8 antibody (1:300, BD Biosciences) in staining solution (0.2% saponin in PBS) for 2 h at RT. After washing the cells with PBS, Hoechst (5 μg/ml, Invitrogen), DY-495-phalloidin (1.2 U/ml, Dyomics) and Alexa Fluor 647-coupled goat anti-mouse IgG (1:400, Invitrogen) were added and incubated for 1 h at RT. The staining procedure was performed using the Biomek NXP Laboratory Automation Workstation (Beckman Coulter).

### *Vaccinia virus*-specific protocol

All liquid handing stages of infection, fixation, and immunofluorescence staining were performed on a liquid handling robot (BioTek, EL406). For infection assays a recombinant WR VACV, WR E EGFP/L mCherry, was utilized. For infection, media was aspirated from the RNAi-transfected cell plates and replaced with 40 μl of virus solution per well (moi = 0.125). Screening plates were incubated for 1 h at 37°C to allow for infection, after which virus-containing media was removed and replaced with 40 μl DMEM/10% FCS. 8 h after infection 40 μl of DMEM/10%FCS containing 20 μM cytosine arabinoside (AraC) was added to all wells to prevent virus DNA replication in secondary infected cells. 24 h after infection cells were fixed by the addition of 20 μl 18% PFA for 30 min followed by two PBS washes of 80 μl. For immunofluorescence staining of EGFP, cells were incubated for 2 h in 30 μl primary staining solution (0.5% Triton X-100, 0.5% BSA, PBS) per well, containing anti-GFP antibody (1:1000). Cells were washed twice in 80 μl PBS, followed by the addition of 30 μl secondary staining solution (0.5% BSA, PBS) containing Alexa Fluor 488 secondary antibody (1:1000), Hoechst (1:10000), and DY-647-phalloidin (1:1200, Dyomics). Cells were washed twice with 80 μl PBS after 1 h incubation in secondary staining solution followed by the addition of 80 μl H_2_O.

### Microscopy

Microscopy was performed with Molecular Devices ImageXpress microscopes. We used the MetaXpress plate acquisition wizard with no gain, 12 bit dynamic range, 9 sites per well in a 3×3 grid with no spacing and no overlap and laser-based focusing. Channels were assay specific (see Additional file 1: Table S2). Robotic plate handling was used to load and unload plates (Thermo Scientific). The objective was a 10X S Fluor with 0.45NA. The Site Autofocus was set to “All Sites” and the initial well for finding the sample was set to “First well acquired”. Z-Offset for Focus was selected manually and “AutoExpose” was used to get a good exposure time. Manual correction of the exposure time was applied to ensure a wide dynamic range with low overexposure, when necessary.

### Statistical analyses

#### Image analysis and data normalization

Image analysis and data normalization was based on modified CellProfiler [28] workflows. Please refer to Additional file 1 for detailed description of computational infrastructure, image analysis, and data normalization.

### Parallel Mixed Model (PMM)

We denote the readout of siRNA *s* silencing gene *g* for a pathogen *g* as *y*_*pgs*_. The linear mixed model of PMM is defined as the following linear model


where *μ*_*p*_ is the fixed effect for pathogen *p* (typically close to 0 because of data Z-Scoring)*, a*_*g*_ is the gene effect overall pathogens, *b*_*pg*_ is the gene effect within a pathogen and *ϵ*_*pgs*_ denotes the error term. The parameters are estimated by maximizing the restricted maximum likelihood using the Newton–Raphson algorithm [33]. We used the implemented version in the “lmer” function from the “lme4” R-package [34]. This implementation allows also the use of weights, which are incorporated by a weighted maximum likelihood formulation. The weights are constant values where each constant corresponds to exactly one data point. For our data, each weight is associated with a single readout of an independent siRNA. The size of the weight indicates the precision of the information contained in the associated readout. The assumptions of the linear mixed model are fulfilled (see Additional file 1: Figure S11).

### Local false discovery rate (q) estimation in PMM

The observed distribution of the estimated *c*_*pg*_ is a mixture of the null *f*_*0*_ and the non-null distribution *f*_*1*_. The null distribution describes the distribution of all genes that are no-hits. The non-null distribution corresponds to the genes that are hits, having either a positive or negative effect. The two distributions are assumed to differ only in the mean. The non-null distribution is shifted by a factor *θ* away from zero. With this we define the local false discovery rate as


where *π*_*0*_ = proportion of true hits and *π*_*1*_ *= 1 - π*_*0*_[59]. The three quantities needed for the estimation of the false discovery rate, are estimated separately by using Maximum Likelihood, Poisson regression, and moment estimation. The estimation procedure is implemented in the function “locfdr” from the “locfdr” R-package [35].

### Data resampling to show that parallel screens add power

We chose gene *g* and pathogen *p* for which we wanted to show the increase in power by simultaneously using the parallel screening structure. In our case, we repeated the analysis for three different cases, consisting of a unique hit (*g: MET, p: L. monocytogenes*), a shared hit (*g: MTOR, p: Vaccinia virus*) and a non-hit *(g: ALK, p: B. abortus*). Each time we resampled data for a fixed number of siRNAs (*n*_*s*_ *= 2,…,11*) and a fixed number of pathogens (*n*_*p*_ *= 2,…,8*) from the full dataset. In detail, we chose randomly (*n*_*p*_*– 1*) pathogens and added additionally pathogen *p*. In the next step, we sampled *n*_*s*_ siRNA sets from the full available set of siRNAs for every gene within all sampled pathogens. We applied PMM on the sampled data and we reported the rank of gene *g* within pathogen *p*. This was repeated 1000 times for each combination of *n*_*s*_ and *n*_*p*_. As a last step we calculated for each combination the mean and variance of the rank for gene *g* within pathogen *p*. For the resampling we omitted genes that have less than 6 siRNA sets, in order to have a good resampling basis. Moreover, we applied the same procedure for the case of *n*_*p*_ *= 1* using MTT.

### Stability analysis

We resampled with replacement 1000 datasets from the full data, taking for each gene the same number of siRNAs as in the full dataset. For each resampled dataset, PMM, MTT and RSA were applied and the corresponding ranking saved. For PMM the ranking was done according to the absolute value of the estimated *c*_*pg*_ effects, for MTT we used the absolute values of the estimated mean and for RSA the ranking based on the *log(p)* values. We took absolute values to take into account down and up hits simultaneously. From the 1000 rankings we calculated the number of genes that appear with high probability (*prob > 0.9 and prob > 0.7*) in the top *k* (*k = 1,…,50*) of the ranking.

### Hit overlaps examined by cross-validation

For the hit cross-validation analysis we only used data coming from the siRNAs of all unpooled libraries (11 in total). In each run, we ran PMM, MTT, and RSA on a subset of the data consisting of 10 individual siRNAs and used the remaining siRNA set as test set. For PMM we ranked the results according to the absolute value of the estimated *c*_*pg*_ effects, for MTT we did ranking with respect to the absolute values of the estimated mean, for RSA we based the ranking based on the *log(p)* values and for the test set we ordered the genes by the absolute value of infection score. We counted the number of genes that appeared in top *k* (*k = 1,…,100*) in both the training and test sets. We determined the counts separately for each pathogen and averaged them in the end.

### Data simulation and ROC-curves

We simulated data by generating 1000 normally distributed screens (mean = 0, std = 0.5) for eight pathogens, taking 4 siRNAs each. Hits were incorporated in the simulated screens by randomly selecting about 10% of the genes (80 out of 826) and shifting them away from zero. The shift was determined by a uniformly distributed random variable. We used the interval [*0.2,0.3*] as parameter for the uniform distribution for “low shift” and the interval [*0.4,0.5*] for “high shift”. We distinguished between three cases: In the first case the hits were different for each pathogen (80 unique hits per pathogen), in the second case all hits were shared between the pathogens (same 80 hits for all pathogens) and in the third case we generated mixed hits (20 unique hits, 20 hits shared between two pathogens, 20 hits shared between four pathogens and 20 hits shared between all eight pathogens). PMM, MTT, and RSA were applied to the simulated data and the ranking was saved. For PMM the results were ranked according to the absolute value of the estimated *c*_*pg*_ effects, for MTT the ranking was done with respect to the absolute values of the estimated mean and for RSA the ranking based on the *log(p)* values. For every ranking list we counted the number of true positives, true negatives, false positives and false negatives in the top *k* (*k = 1,…,826*) and computed the true positive rate (TPR = FP/(FP + TN)) and the false positive rate (FPR = FP/(FP + TN)).

### Influence of parallelism

For selected pathogen *p* we generated 1000 new datasets by fixing the data of *p* and randomizing the data of the other 7 pathogens. We applied PMM to each dataset and saved the resulting ranking of *p*. In the next step we aggregated the 1000 rankings by taking the average over the *c*_*pg*_ scores. We compared the averaged scores to the gene rankings obtained by PMM performed using the original dataset. We independently performed the study for each pathogen.

### Availability of supporting data

The data sets supporting the results of this article are available on the InfectX openBIS data publication portal, that is located at http://www.infectx.ch/dataaccess/. The visitor username is “rdgr2014“ and the corresponding password is “IXPubReview”. The R-package PMM and related documentation is also available on this page.

## Electronic supplementary material

Additional file 1: **Supplementary Information.** The additional data file 1 contains supporting information und further analysis results. (PDF 2 MB)

## Abbreviations

FDR: False discovery rate
GSEA: Gene set enrichment analysis
MTT: Moderated T-test
PMM: Parallel mixed model
ROC: Receiver operating characteristic
RSA: Redundant SiRNA Activity.
