# Supplementary material for: Simultaneous analysis of large-scale RNAi screens for pathogen entry
Source: BMC Genomics. 2014 Dec 22;15(1):1162. doi: 10.1186/1471-2164-15-1162 (PMC4326433; doi:10.1186/1471-2164-15-1162)

Supplementary information

Table of contents

InfectX 2
Assays performed 2
Differences in protocols 2
Quality control 3
Computational infrastructure 4
openBIS 4
Cluster computing and data storage 4
Image analysis 4
Object detection 4
Feature extraction 5
Infection detection and measurement 5
Data preprocessing and normalization 8
Prediction of siRNA on-target genes 8
Z-Scoring 8
Dependency of infection index to population context 8
Cell number readout reproduces conclusions data reproducibility 9
Data fulfills requirements for Moderated T-Test 9
Study of parallelism 9
Reference methods (RSA and MTT) 9
Comparing the results of PMM, MTT and RSA 10
Comparative GSEA results 10
PMM results for cell number readout 10
Data fulfills requirements for PMM 10
Supplementary references 11
Figure Legends 13
Supplementary Figures 15

InfectX

InfectX is a Switzerland-based consortium consisting of eleven research groups in the framework of SystemsX.ch, the Swiss initiative for systems biology. Its focus is on comparatively studying the infection processes of bacterial and viral pathogens and developing computational methods for image analysis and statistical modeling. InfectX has a strong emphasis on developing and using unified wet-lab and analysis protocols and workflows. Supplementary Figure 1 shows the outline of the analysis workflow.

Assays performed

We screened all studied pathogens with four different siRNA libraries (Dharmacon pooled, Dharmacon unpooled, Ambion unpooled, and Qiagen unpooled). Supplementary Table I reports the number of replicates of different pathogen assays. The value in parenthesis indicates the number of assays that needed to be removed because of different problems (e.g. transfection did not work or an older protocol was used that gave a weak fluorescent signal). We used the Dharmacon pooled library for assay optimization and several of them needed to be removed. In all analyses we averaged data from replicates in order to have only one value per library and gene.

Supplementary Table I: Replicates performed for different pathogens and libraries.

Pathogen	Dharmacon 1 pooled siRNA	Ambion 3 unpooled siRNAs	Qiagen 4 unpooled siRNAs	Dharmacon 4 unpooled siRNAs
Adenovirus	7 (+1)	2	1	1
B. abortus	7 (+1)	2	1	2
B. henselae	4 (+1)	3 (+1)	1 (+1)	1
L. monocytogenes	4	2 (+2)	1	1
Rhinovirus	6 (+2)	2	1	1
S. flexneri	4 (+2)	2	1	1
S. typhimurium	3 (+4)	3	1	1
Vaccinia virus	1 (+1)	2	1	1

Differences in protocols

We designed all pathogen specific protocols so that the resulting phenotypes (infection index and cell number) would end up as similar as possible between all the screens. We aimed to reach a cell number for wells where there would be some empty space between the cell colonies (meaning roughly 1500 cells the imaged area of a well). In addition, we aimed to reach infectivity close to 30-50% in a typical well. This would enable the reliable detection of both down and up hits in the infection index readout. However, due to biological issues, sometimes we could not reach these goals, for example for *B. abortus* the typical infection rate remained low (typically close to 5%) and for *B. henselae* the infection rate was high (typically close to 90%). The different infection biology of different pathogens required adaptations to the protocols in order to aim to the above mentioned goals. Supplementary Table II summarizes the main differences in protocols.

Supplementary Table II: Table of pathogens and main differences in protocols.

Pathogen	Strain	Seeded cell number/well	Multiplicity of infection (MOI)	Pathogen entry time (pi: primary infection; si: secondary infection)	Total infection time	DNA stain	Actin stain	Pathogen detection	Additional stain
Adenovirus	Ad2_ΔE3B-eGFP	700	0.1	16 h	16 h	DAPI	Cy5: DY-647-phalloidin	GFP	
B. henselae	*B. henselae* ΔbepG	300	400	30 h	24 h	DAPI	RFP: DY-547-Phalloidin	GFP + Actin	Cy5: Alexa 647
B. abortus	*B. abortus* 2308 pJC43 (*aphT::GFP*)	500	10000	4 h	44 h	DAPI	RFP: DY-547-phalloidin	GFP	-
L. monocytogenes	EGD-e.PrfA* GFP	600	25	1 h	5 h	DAPI	Cy5: DY-647-phalloidin	GFP	0.2% saponin, PBS containing Alexa Fluor-647 coupled anti-rabbit antibody, Cy3: InlC
Rhinovirus	HRV1a	1000	8	7 h	7 h	DAPI	Cy5: DY-647-phalloidin	GFP	Anti-VP2 antibody Mab 16/7 followed by anti-mouse IgG coupled to Alexa Fluor 488
S. typhimurium	S.TmSopE_pM975, SL1344, sopE2sipAsopB (S. TmSopE pM975)	550	80	20 min	4 h	DAPI	RFP: DY-547-Phalloidin	GFP	-
S. flexneri	*S. flexneri* M90T ΔvirG pCK100	600	15	30 min	3.5 h	Hoechst	GFP: DY-495-Phalloidin	DsRed	Anti-mouse IpG coupled to Alexa Fluor 647, Cy5: IL-8
Vaccinia virus	WR E EGFP/L mCherry	600	0.125	1 h (pi), 8 h (si)	24 h	Hoechst	Cy5: DY-647-phalloidin	GFP, RFP	Anti-GFP antibody followed by Alexa Fluor 488

Quality control

Quality control is an umbrella term that covers many different approaches and methods to guarantee "correctness" and "unbiasedness" of the data. Since a large-scale high-content screening setup includes many different kind of steps (wet-lab, imaging, data storage, image analysis and statistics - just to name a few), systematic quality control is essential. The main goal is to control known problem types and tackle them by appropriate approaches in each step. In addition, some a-priori unknown problems can be discovered by clever quality control and automated analysis. However, it is hard to be aware of all possible error sources and therefore we may not be able to check the data against all of them. For complex high-throughput and high-content data the number of possible known error types is usually very large. Therefore, we created standard operating procedures (SOPs) for quality control in order to systemitically go through them and ensure that our

data does not contain any of these known problem types. InfectX defined one common Quality Control SOP for all pathogens. We also clearly defined the responsible person for each task.

Computational infrastructure

We have identified data analysis and computational infrastructure to be key aspects of attaining reproducible high-content screening results. Small variations in analysis procedures or the software environment can lead to differences in numerical results or file formats. These incompatibilities are hard to spot at the time they arise, but might significantly increase the effort or even invalidate comparable data analysis from varying sources. InfectX has made considerable efforts to ensure that the data is analyzed in a reproducible and accessible way. In more detail, key aspects of our data analysis are: all results are annotated with the methods and settings that were used in their creation new methods undergo regression testing before being applied to any dataset. Result datasets are automatically shared through our openBIS data portal with all members of the consortium.

openBIS

Data management and data sharing was performed using the openBIS biology information system [1]. To this end, openBIS has been extended to support screening metadata like the siRNA library, and screening results like images, well-based readouts and object-based features. For example, one extension includes the visualization of images together with their analysis results.

iBRAIN2: workflow and process management for screening data

In response to the data analysis requirements of InfectX, an open-source workflow management solution called iBRAIN2 was developed [2]. (http://ibrain2.sourceforge.net/). iBRAIN2 can employ openBIS as a data management solution and enables the parallel analysis of HCS datasets on high-performance computing (HPC) clusters. It provides resilient and flexible workflow management capabilities in order to face the number and complexity of the analysis steps performed on these typically large datasets. The modular design of this solution allowed the definition of sequential analysis steps to be performed on acquired data. These workflows are not instance-specific and success/failure criteria can be defined for each sub-step. They can therefore be part of SOPs and simplify quality control during data analysis.

Cluster computing and data storage

Data analysis was performed on a Linux-based computer cluster of heterogeneous multicore nodes running Sun Grid Engine on CentOS Linux. The nodes are based on x86_64 architecture with 2GB RAM per process. About 100.000 CPU hours where used in data analysis. Data storage is performed on an NFS-mounted IBM SONAS storage system.

Image analysis

Object detection

The following steps are common for all pathogens. Images were first scaled so that pixel intensities of a full plate are in the 0 to 1 range. Images were then corrected for shading (flat field correction, vignetting correction) by applying a shading model to the image pixels. Shading-corrected images were stored in floating points to reduce the loss of information. For bacterial pathogens *B. henselae*, *B. abortus*, and *S. flexneri*, the pathogen signal in the DAPI channel (referring to both DAPI and Hoechst stainings) was removed to increase the quality of the nucleus segmentation. The pathogen signal was removed by subtracting a linear transformation of the GFP channel (referring to pathogen specific infection channel) from the DAPI channel. After the pathogen signal reduction, DAPI images

were stored in double precision to reduce loss of information. On the corrected images, object detection was performed using CellProfiler [3]. First, nucleus objects labeled “Nuclei“ were segmented in the DAPI channel using OTSU’s method (CellProfiler module IdentifyPrimAutomatic). Second, a peri-nuclear ring object labeled “PeriNuclei” was constructed by extending the nucleus object by eight pixels and removing the nuclear area from the so extended nuclear area (CellProfiler modules ExpandOrShrink and IdentifyTertiary). Third, a cell body object labeled “Cells” was segmented in the Actin channel using the “Propagation” method around the nucleus object (CellProfiler module IdentifySecondaryInformed). Fourth, a non-actin based cell body object labeled “VoronoiCells” was constructed by extending the nucleus object by twenty-five pixels (CellProfiler module ExpandOrShrink).

Feature extraction

The following steps were common for all pathogens. On the segmented objects, measurements were performed using CellProfiler. On all four segmented objects (Nuclei, PeriNuclei, Cells, VoronoiCells) shape measurements were extracted. Intensity and texture measurements were extracted with respect to all available channels (DAPI, Actin, Pathogen, and pathogen-specific channel where applicable). The neighborhood relationship was measured for cell body objects that are within a two-pixel distance of each other. All measurement result files of CellProfiler were stored in the openBIS database alongside the original images. For ease of access, the data was refactored so that only one class of measurements is contained in a single unique file with the same internal structure as the original CellProfiler result file.

Infection detection and measurement

The approach for detection of infected cells in images was pathogen specific (details for each pathogen are listed below). The result of the infection detection was for all pathogen assays a cellular phenotype that indicates infection of an individual cell on a binary level (the cell is infected vs. the cell is not infected). In addition, some of the infectious phenotypes indicate the level of infection for each cell. The binary infection phenotype allows us to define the infection index readout for all pathogens. The infection index is defined as: number of infected cell / total number of cells in the well. The infection index is the main readout for all pathogens. Most of the pathogen screens also include additional stains for other pathogen infection related phenotypes (secondary readouts). These additional phenotypes are not discussed or analyzed in detail in this paper. We used several different algorithms to detect the binary infection phenotypes (i.e. infection scoring). We found that for quality control it is important to use different methods of infection scoring. Deviations in the infection score readouts point to possible problems and agreements between the readouts cross-validate the various infection scoring methods. For all pathogens, with the exception of *B. henselae*, we used at least two of the following infection scoring algorithms for infection detection. For *B. henselae* we applied a separate algorithm because of its special infection phenotype in form of invasomes (see *B. henselae* infection detection and measurement).

Decision Tree Infection Scoring (DTIS)

We selected a small number of image analysis single cell features that were most sensitive to the infection phenotype (typically from two up to five features). The N features are evaluated in a decision tree, which is a complete binary tree with N levels and 2^N nodes. Each node is evaluated by applying a threshold to the corresponding feature. During traversal of the tree, if the feature exceeds the threshold, evaluation continues with the one child, and if the feature does not exceed the threshold, evaluation continues with the other child. Nodes of the lowest level connect to one of the

two distinct end states “infected” and “uninfected”. The connection of the nodes to children and the choice of features are performed once by an expert and remain static for all plates of a pathogen. The choice of the decision tree thresholds is affected by plate-specific parameters like quality of the staining, cell vitality and microscope illumination, and must be adjusted on a plate-by-plate basis. We supply a table that lists for each plate the used features and their corresponding thresholds.

SVM infection scoring

We used CellClassifier [4] and supervised machine learning using a Support Vector Machine based binary classifier [5] to separate infected cells from non-infected cells. Most pathogens show a clear binary infection phenotype for cells (for example *S. typhimurium* or *Vaccinia virus*). For these screens, the supervised machine learning infection scoring was relatively straight-forward and the Support Vector Machine based classifier typically produced high quality results (classification accuracy >99%). However, some pathogens have relatively continuous infections levels (for example *L. monocytogenes*). For these screens, supervised binary classification was not optimal and subjective evaluation (e.g. to decide which cell is infected enough to be classified as infected) was required during the training phase. For all pathogens 3 to5 features were manually selected and the features were plate-wise Z-Scored prior to applying SVM learning.

Segmentation based infection scoring

For some pathogens we can apply image-based segmentation of pathogen objects in CellProfiler to detect pathogen colonies or single pathogens in the cell. Therefore, we used a segmentation method based on the OTSU method or on wavelets. Pathogen object segmentation leads directly to a binary infection scoring for each cell. A cell is defined as “infected” if a pathogen object overlaps mostly with this cell. This definition ensures that no pathogen object is considered belonging to more than one cell, even though if it overlaps with more than one cell.

Adenovirus infection detection and measurement

Cell were infected with a replication competent Ad2_GFP_dE3B (in short *Adenovirus*) [6]. Infected cells were scored by their dispersed GFP signal of variable intensity across the cell body, most prominently in the nuclear area. Strength of the signal was strictly dependent on the amount of virus added to the cells, ranging from very strong intensity (high infection) to background intensity (no or very low infection). To quantify *Adenovirus* infection, GFP intensity was measured in the objects Nuclei, PeriNuclei, Cells and VoronoiCells using CellProfiler module MeasureObjectIntensity

B. henselae infection detection and measurement

For *B. henselae* screens, infection of a cell is defined by the appearance of pathogen-induced invasome structure, an Actin surrounded membrane structures containing a large bacterial aggregate internalized as a whole in the cell body. Invasome object detection was performed by applying template matching to the Actin channel using templates of an idealized invasomes of varying size. A segmentation of the invasome was achieved by determining the sphere of maximum actin intensity surrounding the candidate location. On the segmented object intensity measurements in the Actin and GFP channel were extracted using CellProfiler module MeasureObjectIntensity. Shape measurements were extracted using module MeasureObjectAreaShape. The invasome detection algorithm was very sensitive to invasome candidates, but also detects false positive invasome structures. We trained classifier based on a Support Vector Machine to separate true invasomes from false positive invasomes. We found the most descriptive feature for true invasomes to be the GFP

channel intensity, which indicates presence of a bacterial cluster. The classification was based on the information in the GFP channel. After the true invasomes were detected, a cell was classified as infected if the cell has one or more true invasomes assigned [7].

B. abortus infection detection and measurement

B. abortus infection appears as large micro colonies across the cell body. To quantify infection for *B. abortus*, GFP intensity was measured in the objects Nuclei, PeriNuclei, Cells and VoronoiCells using CellProfiler module MeasureObjectIntensity.

L. monocytogenes infection detection and measurement

L. monocytogenes infection appears as dispersed Cy3 signal of varying intensity across the cell body. The strength of the signal is dependent on the amount of bacteria in the cell, ranging from very strong intensity (high infection) to background intensity (very low to no infection). The majority of signal is accumulated in the perinucleus. To quantify infection for *L. monocytogenes*, Cy3 intensity was measured in the objects Nuclei, PeriNuclei, Cells and VoronoiCells using CellProfiler module MeasureObjectIntensity.

Rhinovirus infection detection and measurement

Cells were infected with the strain HRV1A as described [8]. The infection phenotype were small cytoplasmic clusters of viral replication sites of varying size detected by the monoclonal antibody 16-7, and a secondary anti-mouse IgG conjugated to Alexa488. Wavelet-based object detection was used to segment the replication objects, and infection was measured by determining the fluorescence intensity using the CellProfiler module MeasureObjectIntensity.

S. flexneri infection detection and measurement

S. flexneri infection appears in form of an accumulation of micro colonies which is often localized in the vicinity of the nucleus (perinucleus). The accumulation is formed because we use a non-motile mutant of *S. flexneri* (ΔvirG), which is not able to move by actin-based motility. Once intracellular, the bacteria replicate themselves resulting in the formation of micro colonies. A cell can contain one or several micro colonies. Extracellular bacteria are not visible as only intracellular bacteria express the fluorescent marker. Segmentation using OTSU's method was used to segment bacteria objects. To quantify infection for S. flexneri, RFP intensity was measured in the bacteria objects using CellProfiler module MeasureObjectIntensity.

S. typhimurium infection detection and measurement

S. typhimurium infection appears as small GFP dots in the cell body. Wavelet-based object detection was used to segment virus objects. To quantify infection for S. typhimurium, GFP intensity was measured in the virus objects using CellProfiler module MeasureObjectIntensity.

Vaccinia virus infection detection and measurement

Vaccinia virus infection appears as dispersed GFP signal of varying intensity across the cell body. To quantify infection for *Vaccinia virus*, GFP intensity was measured in the objects Nuclei, PeriNuclei, Cells and VoronoiCells using CellProfiler module MeasureObjectIntensity.

Data preprocessing and normalization

Prediction of siRNA on-target genes

A target gene for a specific siRNA is defined as a gene, which exhibits perfect complementarity within its coding region to this siRNA. This is not necessarily a 1:1 relation, i.e. siRNAs can potentially have multiple target genes. In order to identify these target relations, siRNA sequences were searched against genomic transcript sequences from RefSeq (release hg19, downloaded 17.07.2012) and ENSEMBL (release GRCh37.67, downloaded 20.07.2012) using BLAST version 2.2.27. The BLAST parameter word_size was set to 7. Transcript matches shorter than siRNA sequence length, as well as matches with gaps were removed. Finally, transcript IDs were translated to gene IDs and the unique set of target gene ID(s) considering both genomic data sources were reported for each siRNA.

Z-Scoring

Individual plates of screens cannot always be handled identically in the wet-lab. For this reason, we often observe differences in the readout levels of single plates or in plate batches. There are several approaches in the literature to correct for these differences [9]. Negative controls (MOCK and SCRAMBLED) sometimes show non-typical phenotypes (such as relatively high cell number) and good positive controls were not always available for all pathogens before primary screening. Therefore, we chose non-control based data normalization methods. We used Z-Scoring to normalize variations between plates as:

$$x_{new} = \frac{x_{old} - \mu}{\sigma}.$$

Here μ is the mean of all siRNA well readouts in the plate, σ is the standard deviation of all siRNA well readouts in the plate, x_{old} is the raw well readout and x_{new} is the normalized well readout. The non-control based normalization assumes that all genes are randomly distributed among all plates and that there are relatively few positive phenotype genes in the whole screen. After the plate Z-scoring we also Z-scored the whole screen in order to generate comparable screens. We used the above mentioned method with the mean and standard deviation of the whole screen.

For Z-Scoring, we need to assume that the data is approximately Gaussian distributed and that only a relatively small number of data points are outliers in each plate. Supplementary Figure 2 shows a histogram of the data and QQ-plot for *the Adenovirus* Dharmacon pooled screen for an example plate. The infection indices are nearly Gaussian distributed. Only a minority of data points in the plate are outliers. Therefore, the assumptions are fulfilled in a good approximation. The results for all the other plates and pathogens are qualitatively similar.

Dependency of infection index to population context

Infection phenotypes can depend on the population context, such as the total cell number [10-12]. Supplementary Figure 3 shows examples that for some screens there is a slight dependency on the cell number (for example *B. henselae*, *L. monocytogenes*, and *Vaccinia virus*), but for some pathogens (*B. abortus*) the dependency is not visible. We conjecture that the HeLa ATCC cell line shows lesser dependency of phenotypes to population context than some other cell lines reported in the literature.

To reduce the bias caused by the correlation we applied non-parametric regression correction with the Lowess-method [13]. To normalize the Z-Scored infection index, we use a sliding window of size 200 to go through the ranked cell number readouts. For each window we calculate the mean *m* and

standard deviation *s*. With those values we then Z-Score the infection index of the well in the center of the window. The Lowess-normalized value is

$$x_{new} = \frac{x_{old} - m}{s}.$$

This method also normalizes the possible biases in standard deviation.

Cell number readout reproduces conclusions data reproducibility

Figure 2 shows data correlations of replicate screens and screens performed with different siRNAs targeting the same genes using the infection index readout. Supplementary Figure 4 shows the results of the same analysis using the cell number readout. Identical qualitative conclusions as for the infection index readout can also be drawn from the cell number readout results.

Data fulfills requirements for Moderated T-Test

The Moderated T-Test (MTT) assumes that the sample standard deviations of the siRNAs within a gene are Chi-squared distributed. This assumption is tested by plotting the observed quantiles of the sample standard deviation versus a Chi-Squared distribution (Supplementary Figure 5a). The points show a straight line for all eight pathogens, indicating that our data satisfies these assumptions. MTT is followed by Storey's multiple testing correction. This approach assumes that the p-values have a flat distribution with a possible peak at the low end. The histograms illustrates that our p-values fulfill this assumption for all pathogens (Supplementary Figure 5b).

Study of parallelism

In the main text we presented a study how parallelism affects the ranking of genes in individual screens when using PMM. In the main Figure 6 we only used *L. monocytogenes* as an example. Supplementary Figure 6 shows the same results for all the pathogens. In all cases we mostly gain hits by including parallel screens into the simultaneous analysis.

Reference methods (RSA and MTT)

As a reference method, we used Moderated T-Test (MTT) [14]. It tests whether the observed distribution of a sample (in our case the collection of readouts of one gene) has a mean equal to 0. In contrast to the one-sample t-test, the test statistic includes as prior information the different variances of the siRNAs within the genes. Therefore, it assumes that the standard deviation of the test samples are chi-squared distributed. We performed MTT using the R implementation presented in [15]. Our data satisfies the method assumptions (see Supplementary Figure 5a). The obtained p-values cannot be directly used in large-scale screening because of the problems caused by multiple testing [16-19]. In recent years, several methods have been proposed to control the significance levels with respect to the False Discovery Rate (FDR) and corresponding q-values [16, 17]. We used the method in Storey and Tibshirani (2003). We refer to their paper for the full description and R implementation of the method. The Storey multiple testing correction assumes that the distribution of p-values is flat, with a possible peak at the lower end [17]. Supplementary Figure 5b shows the histogram of the p-values for all pathogens.

The Redundant SiRNA Analysis (RSA) ranks all siRNAs targeting a given gene over all siRNAs in the screens. It assigns the p-values for each gene based on a hypergeometric distribution that indicates whether the distribution of ranks of this gene is shifted significantly towards low ranks [12]. RSA was run using the R-package "RSA" release 1.2 [12] with parameters: *l=-1.5* and *u=1*, where *l* refers to the

threshold where a single siRNA readout is considered to be true positive at the low end and *u* refers to the threshold where a single siRNA readout is considered to be true positive at the high end.

Comparing the results of PMM, MTT and RSA

We compared the results of PMM, MTT and RSA by scatterplots of the gene ranks originating from these methods. In the main text we presented an example of MTT compared to PMM for *L. monocytogenes* (Figure 6b). Supplementary Figure 7 shows the comparisons between all method pairs for all pathogens. The correlations between the three different methods are relatively high. In particular, the top hits are most similar and genes are only slightly shuffled when using different hit ranking methods. Using the same setting as for the calculation of the ROC curves, we also compared missed rates between PMM; MTT and RSA. The results show that the false negative rate of our model is not higher than with the other commonly used methods (see Supplementary Figure 8).

Comparative GSEA results

In order to evaluate the biological relevance of found hits, we calculated pathway enrichment scores separately for each pathogen by the Gene Set Enrichment Analysis (GSEA) algorithm using as input the results from the three hit ranking algorithms PMM, MTT, and RSA. Gene Set Enrichment Analysis (GSEA) was run using the Java-package "gsea2-2.1.0.jar" and the curated canonical pathways "c2.cp.v4.0.entrez.gmt" [20]. The following settings were used for the parameters: collapse was set to false, mode to Max_probe, norm to meandiv, nperm to 1000, scoring_scheme to classic, include_only_symbols to true, make_sets to true, set_max to 500 and set_min to 7. We limited ourselves to pathways that had at least 7 kinases within the pathway in order to avoid bias towards too small pathways. We decided to use the "classic" approach (instead of the recommended "weighted" approach) in order to keep different hit scoring methods (PMM, MTT, and RSA) comparable. We selected all the pathways that were significant (GSEA pathway enrichment FDR score < 0.2) for any pathogen and method pair. The results are illustrated in Supplementary Figure 9. The heatmap shows GSEA pathway enrichment scores for all pathogens using as input the ranked lists of infection index down hits detected by PMM, MTT, and RSA. PMM found more significant pathways than the other methods for most pathogens.

PMM results for cell number readout

We fitted PMM also for the cell number readout. The fitted PMM yielded for all pathogens the same c_{pg} scores (the random effects b_{pg} were estimated to 0) and the same q_{pg} for all genes. Therefore, we obtain the same significant genes for all pathogens (Supplementary Figure 10). The results reflect the fact that cell number is a pathogen independent readout.

Data fulfills requirements for PMM

PMM assumes that the model residuals ε_{pgs}, as well as the random coefficients a_g and b_{pg} are normally distributed. Supplementary Figure 11 shows diagnostic plots for the fitted PMM. The QQ-plot shows that the normal distribution for residuals is only approximately satisfied (Supplementary Figure 11a). There are outliers in the residuals with respect to positive and negative infection indices (marked with red points). To check whether the estimation of the PMM is affected by these outliers, we refitted the PMM without the red marked points. The resulting hit lists of the PMM with and without the red points are almost identical. Moreover, the residuals are randomly distributed around zero for each gene within a pathogen (Supplement Figure 11b–c). This indicates that there is no systematic error in the estimation of the PMM. Therefore, the PMM fit is reliable concerning the

residuals assumptions. The two other QQ-plots confirm the assumption of normal distribution for the random coefficients (Supplementary Figure 11d–e).

Supplementary references

1. Bauch A, Adamczyk I, Buczek P, Elmer FJ, Enimanev K, Glyzewski P, Kohler M, Pylak T, Quandt A, Ramakrishnan C *et al*: **openBIS: a flexible framework for managing and analyzing complex data in biology research**. *BMC Bioinformatics* 2011, **12**:468.
2. Rouilly V, Pujadas E, Hullar B, Balazs C, Kunszt P, Podvinec M: **iBRAIN2: automated analysis and data handling for RNAi screens**. *Stud Health Technol Inform* 2012, **175**:205-213.
3. Carpenter AE, Jones TR, Lamprecht MR, Clarke C, Kang IH, Friman O, Guertin DA, Chang JH, Lindquist RA, Moffat J *et al*: **CellProfiler: image analysis software for identifying and quantifying cell phenotypes**. *Genome Biol* 2006, **7**(10):R100.
4. Ramo P, Sacher R, Snijder B, Begemann B, Pelkmans L: **CellClassifier: supervised learning of cellular phenotypes**. *Bioinformatics* 2009, **25**(22):3028-3030.
5. Hlava VFaV: **Pattern recognition Toolbox for Matlab**. Czech Technical University in Prague; 2000.
6. Yakimovich A, Gumpert H, Burckhardt CJ, Lutschg VA, Jurgeit A, Sbalzarini IF, Greber UF: **Cell-free transmission of human adenovirus by passive mass transfer in cell culture simulated in a computer model**. *J Virol* 2012, **86**(18):10123-10137.
7. Truttmann MC, Guye P, Dehio C: **BID-F1 and BID-F2 domains of Bartonella henselae effector protein BepF trigger together with BepC the formation of invasome structures**. *PloS one* 2011, **6**(10):e25106.
8. Jurgeit A, Moese S, Roulin P, Dorsch A, Lotzerich M, Lee WM, Greber UF: **An RNA replication-center assay for high content image-based quantifications of human rhinovirus and coxsackievirus infections**. *Virol J* 2010, **7**:264.
9. Birmingham A, Selfors LM, Forster T, Wrobel D, Kennedy CJ, Shanks E, Santoyo-Lopez J, Dunican DJ, Long A, Kelleher D *et al*: **Statistical methods for analysis of high-throughput RNA interference screens**. *Nat Methods* 2009, **6**(8):569-575.
10. Snijder B, Sacher R, Ramo P, Liberali P, Mench K, Wolfrum N, Burleigh L, Scott CC, Verheije MH, Mercer J *et al*: **Single-cell analysis of population context advances RNAi screening at multiple levels**. *Mol Syst Biol* 2012, **8**:579.
11. Knapp B, Rebhan I, Kumar A, Matula P, Kiani NA, Binder M, Erfle H, Rohr K, Eils R, Bartenschlager R *et al*: **Normalizing for individual cell population context in the analysis of high-content cellular screens**. *BMC Bioinformatics* 2011, **12**:485.
12. Konig R, Chiang CY, Tu BP, Yan SF, DeJesus PD, Romero A, Bergauer T, Orth A, Krueger U, Zhou Y *et al*: **A probability-based approach for the analysis of large-scale RNAi screens**. *Nat Methods* 2007, **4**(10):847-849.
13. Yang YH, Dudoit S, Luu P, Lin DM, Peng V, Ngai J, Speed TP: **Normalization for cDNA microarray data: a robust composite method addressing single and multiple slide systematic variation**. *Nucleic Acids Res* 2002, **30**(4):e15.
14. Smyth GK: **Linear models and empirical bayes methods for assessing differential expression in microarray experiments**. *Stat Appl Genet Mol Biol* 2004, **3**:Article3.
15. Smyth GK: **Limma: linear models for microarray data**. In: *Bioinformatics and Computational Biology Solutions using R and Bioconductor.* Edited by R. Gentleman VC, S. Dudoit, R. Irizarry, W. Huber. New York: Springer; 2005: 397-420.
16. Storey JD: **A direct approach to false discovery rates**. *Journal of the Royal Statistical Society Series B-Statistical Methodology* 2002, **64**:479-498.
17. Storey JD, Tibshirani R: **Statistical significance for genomewide studies**. *Proc Natl Acad Sci U S A* 2003, **100**(16):9440-9445.
18. Prummer M: **Hypothesis Testing in High-Throughput Screening for Drug Discovery**. *J Biomol Screen* 2012, **17**(4):519-529.

19. Benjamini Y, Hochberg y.: **Controlling the false discovery rate: a practical and powerful approach to multiple testing**. *Journal of the Royal Statistical Society, Series B* 1995, **57**(1):289–300.
20. Subramanian A, Tamayo P, Mootha VK, Mukherjee S, Ebert BL, Gillette MA, Paulovich A, Pomeroy SL, Golub TR, Lander ES *et al*: **Gene set enrichment analysis: a knowledge-based approach for interpreting genome-wide expression profiles**. *Proc Natl Acad Sci U S A* 2005, **102**(43):15545-15550.

Figure Legends

Supplementary Figure 1.

InfectX data analysis workflow. We screened 11 single siRNA libraries (4 siRNAs from Dharmacon, 4 siRNAs from Qiagen, and 3 siRNAs from Ambion) and one pooled library from Dharmacon with 8 pathogens. We performed imaging with Molecular Devices ImageXPress microscopes with 10x magnification with 9 sites per well on 3-4- channels depending on the assay. Image analysis consisted of image shading correction, object segmentation (nuclei, perinuclei, cells, and Voronoi cells), feature extraction (typically 200 features per cell), and infection scoring (with up to three algorithms). We normalized well-based data with plate Z-scoring, population regression (Lowess), and experiment Z-scoring. Technical data aggregation steps were followed by hit detection (PMM, MTT, and RSA) and False Discovery Rate (FDR) analysis. We performed several comparative analyses (method comparison, GSEA enrichment analysis, and STRING network analysis). Data is publicly shared using the openBIS database. All data including raw images, single cell data, assay metadata, and well data are fully accessible through openBIS web GUI and several programming interfaces (APIs).

Supplementary Figure 2.

Data fulfills assumptions for Z-Scoring. (a) Histogram and (b) QQ-plot of non-normalized infection indices from plate 3 of the Adenovirus Dharmacon Pooled screen. The plots show a distribution with slightly fatter tails than Gaussian.

Supplementary Figure 3.

(a) Scatter plots showing dependencies of Z-Scored infection indices to cell number. Red lines correspond to the smoothed Lowess average estimate and green lines to the +/- standard deviation estimates of the dependency. The example data are from the Dharamacon pooled siRNA libraries.

Supplementary Figure 4.

Using more siRNAs adds power to yield reproducible results. (a) The three boxplots show the Pearson correlation coefficient R between screens performed using the same siRNA set. The numbers 1 to 3 correspond to the number of replicate screens that are averaged and compared to another distinct set of replicate screens, averaged over the same number. The replicate screens were resampled 500 times. The scatter plot shows an example for the correlation of cell numbers (CN) from a duplicate of Adenovirus Dharmacon pooled screen. (b) The set of six boxplots show the Pearson correlation coefficients of the averaged readouts from 1 to 6 siRNA sets. The scatter plots depict the correlation of cell numbers for Adenovirus, the first between two different single siRNAs and the second between each an average over six siRNAs.

Supplementary Figure 5.

(a) QQ-plot comparing the observed quantiles of the sample standard deviations of the siRNAs within each gene to a Chi-squared distribution. Different colors represent different pathogen assays. (b) Each line shows the distributions of p-values originating from MTT using as input the infection index readout for one pathogen.

Supplementary Figure 6.

The y-axis shows the estimated cpg scores for the pathogen indicated in the title. The x-axis shows cpg scores originating from a refitted PMM based on data where we randomized the other 7 parallel assays. The colors correspond to hit genes (FDR < 0.4) in different cases: green is a hit in both cases,

red is a hit in the fit based on the original data, and blue is a hit in the fit based on the randomized data.

Supplementary Figure 7.

(a) The y-axis shows the rank of a gene given by PMM and the x-axis the rank defined by MTT. The dot size corresponds to the sharedness score of each gene. The results of each pathogen are plotted separately in each plot. (b) Comparison of the ranks resulting from PMM and RSA for all eight pathogens. (c) Comparison of the ranks resulting from RSA and MTT for all eight pathogens.

Supplementary Figure 8.

The figure shows DET-curves for PMM, MTT and RSA applied on simulated data for three different scenarios (containing only hits that were shared between all pathogens, unique hits for all pathogens and mixed hit structure of both unique and shared hits). The dashed and solid lines indicate whether the shifts were generated by a low or high shift away from zero.

Supplementary Figure 9.

GSEA pathway enrichment results for PMM, MTT, and RSA hit ranking methods. The numbers give the number of highly significant pathways (GSEA FDR < 0.2) for each hit detection method and pathogen. The significant pathways are high-lighted with a red square.

Supplementary Figure 10.

Summary of screening hits for the cell count readout. The heat map shows all genes which were significant (FDR < 0.4) at least for one pathogen. The black outlines indicate significant genes (all the genes were significant for all pathogens) and the green outlines indicate the strongest hit. The colors correspond to the estimated cpg values.

Supplementary Figure 11.

(a) QQ-plot comparing the observed quantiles of residuals from the PMM to the theoretical quantiles of a normal distribution. (b) Scatterplot of residuals of all combinations of genes and pathogens. (c) “Zoomed-in-version” of the scatterplot in (b) at both ends. The plot shows a random scatter around 0, indicating that there are no systematic errors in the estimation of PMM. (d) QQ-plot comparing the observed quantiles of the gene random effects (ag) to the theoretical quantiles of a normal distribution. (e) QQ-plot of the gene random effect within a pathogen (bpg).

Supplementary Figure 1

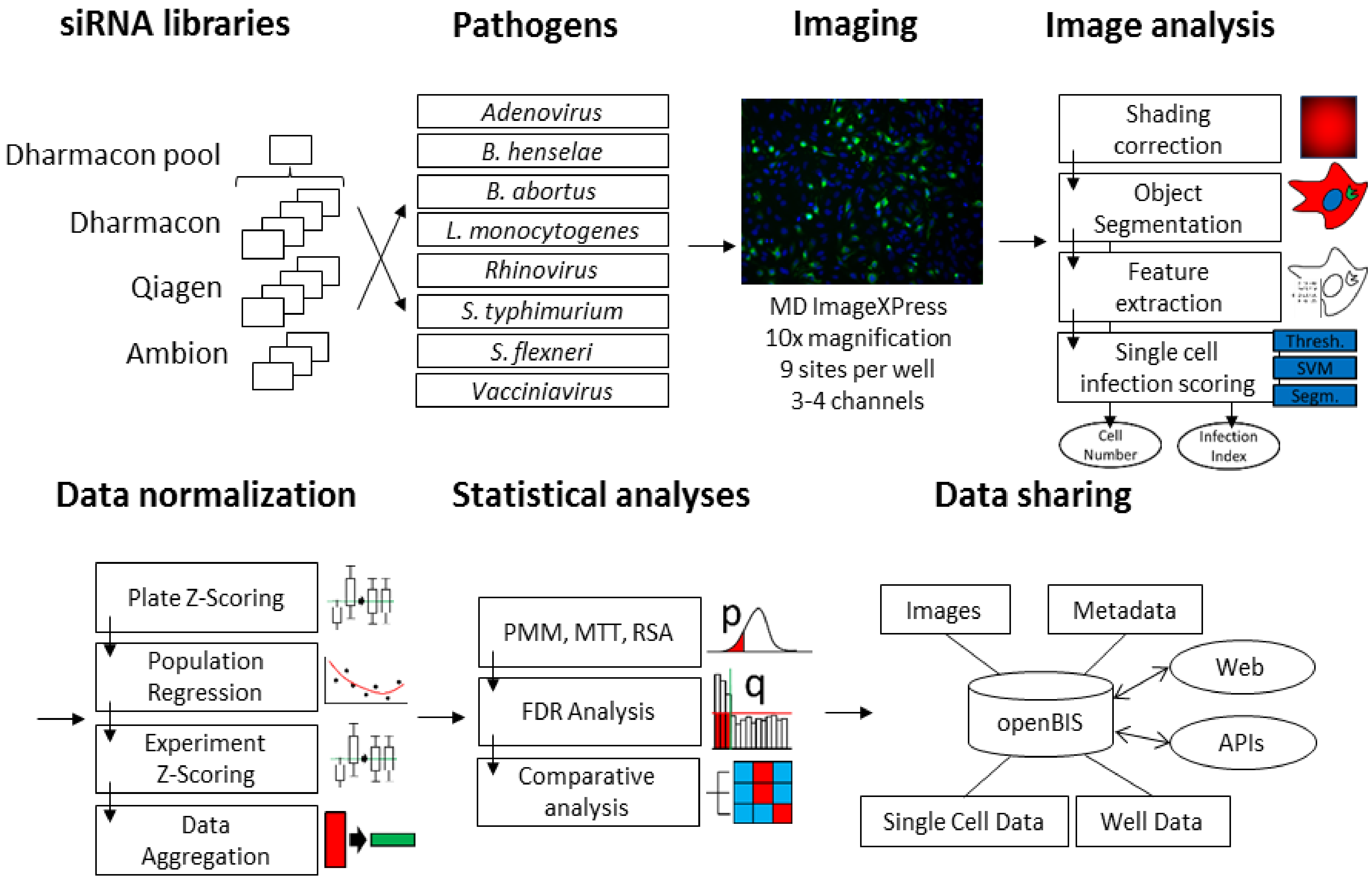

Supplementary Figure 2

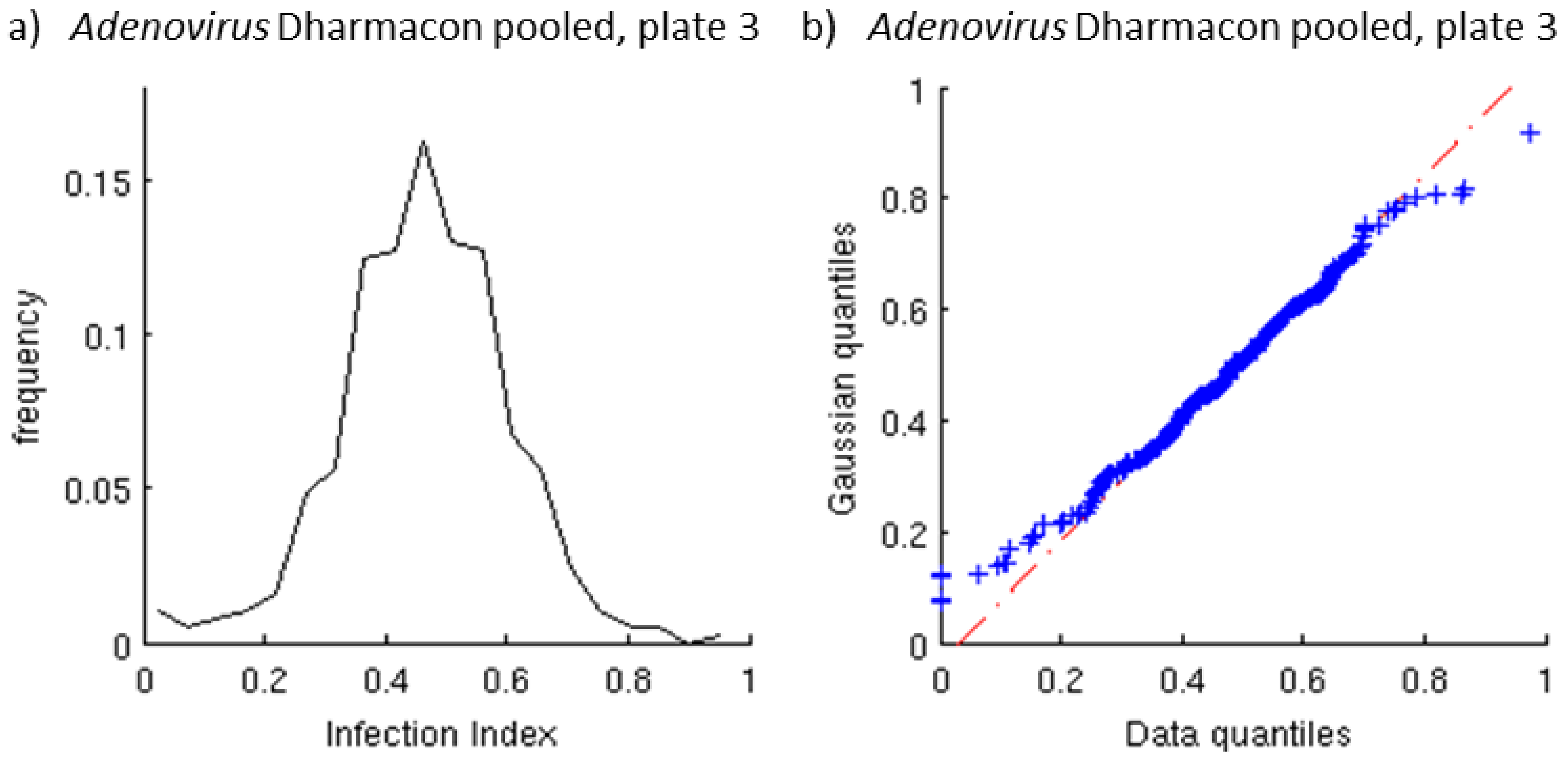

Supplementary Figure 3

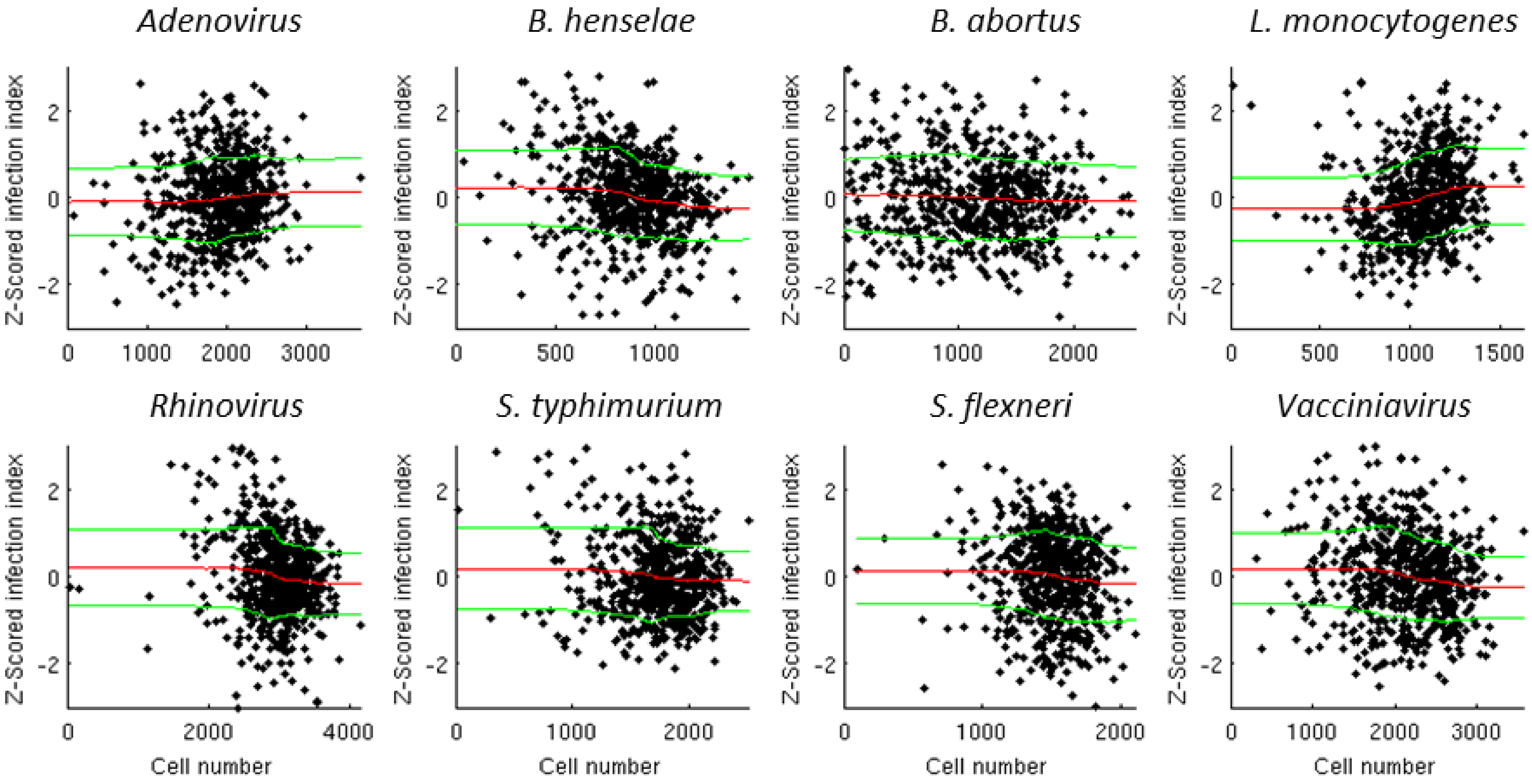

Supplementary Figure 4

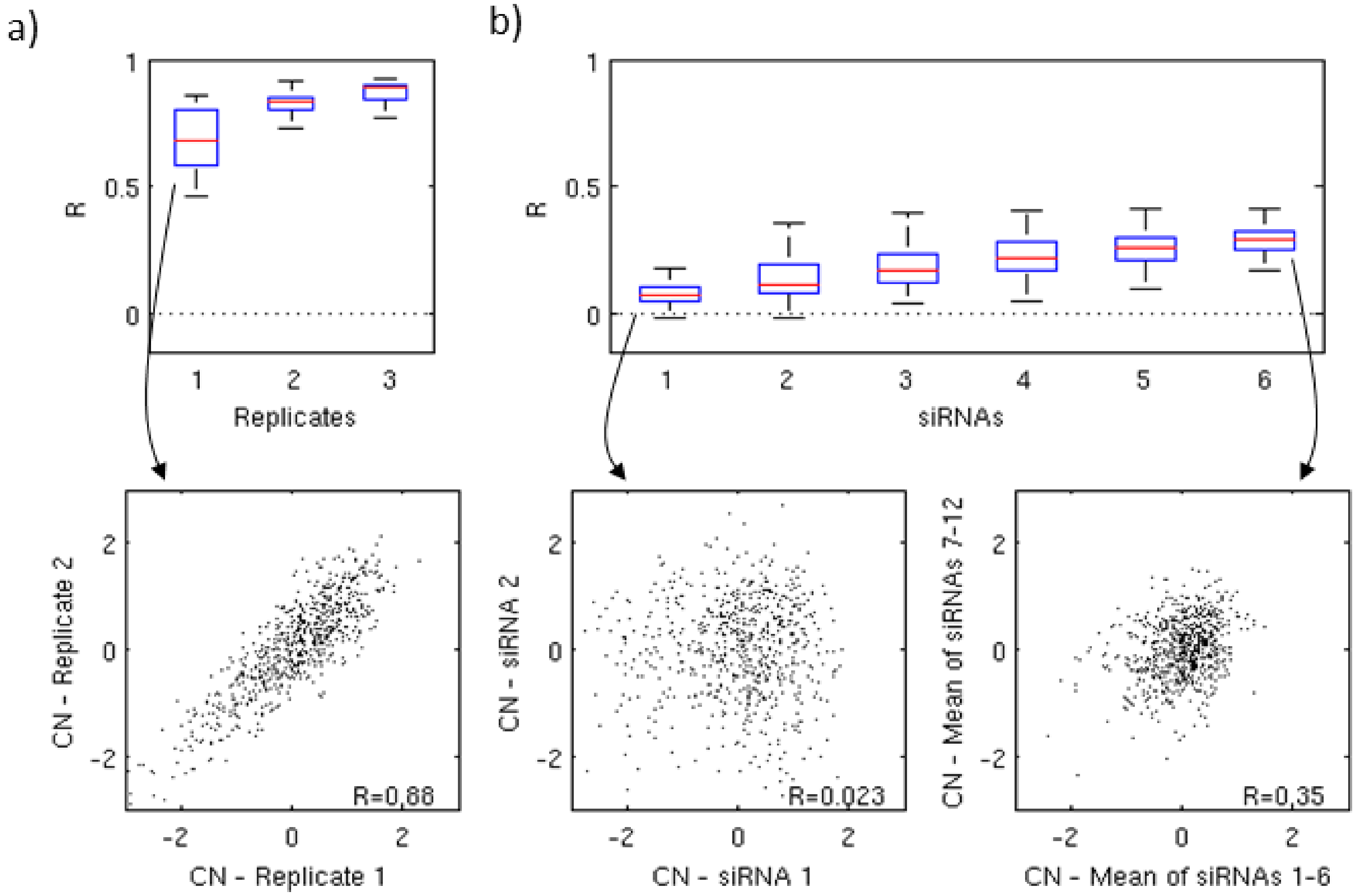

Supplementary Figure 5

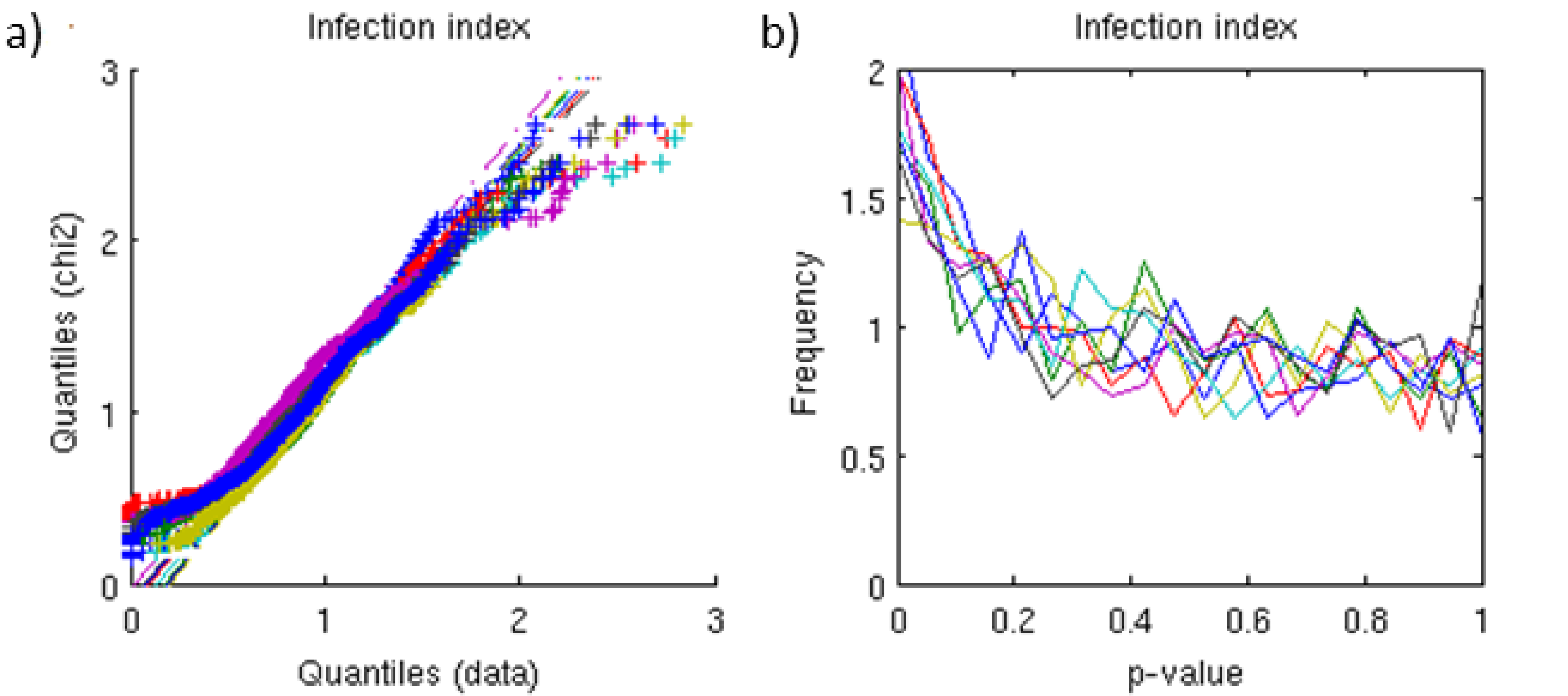

Supplementary Figure 6

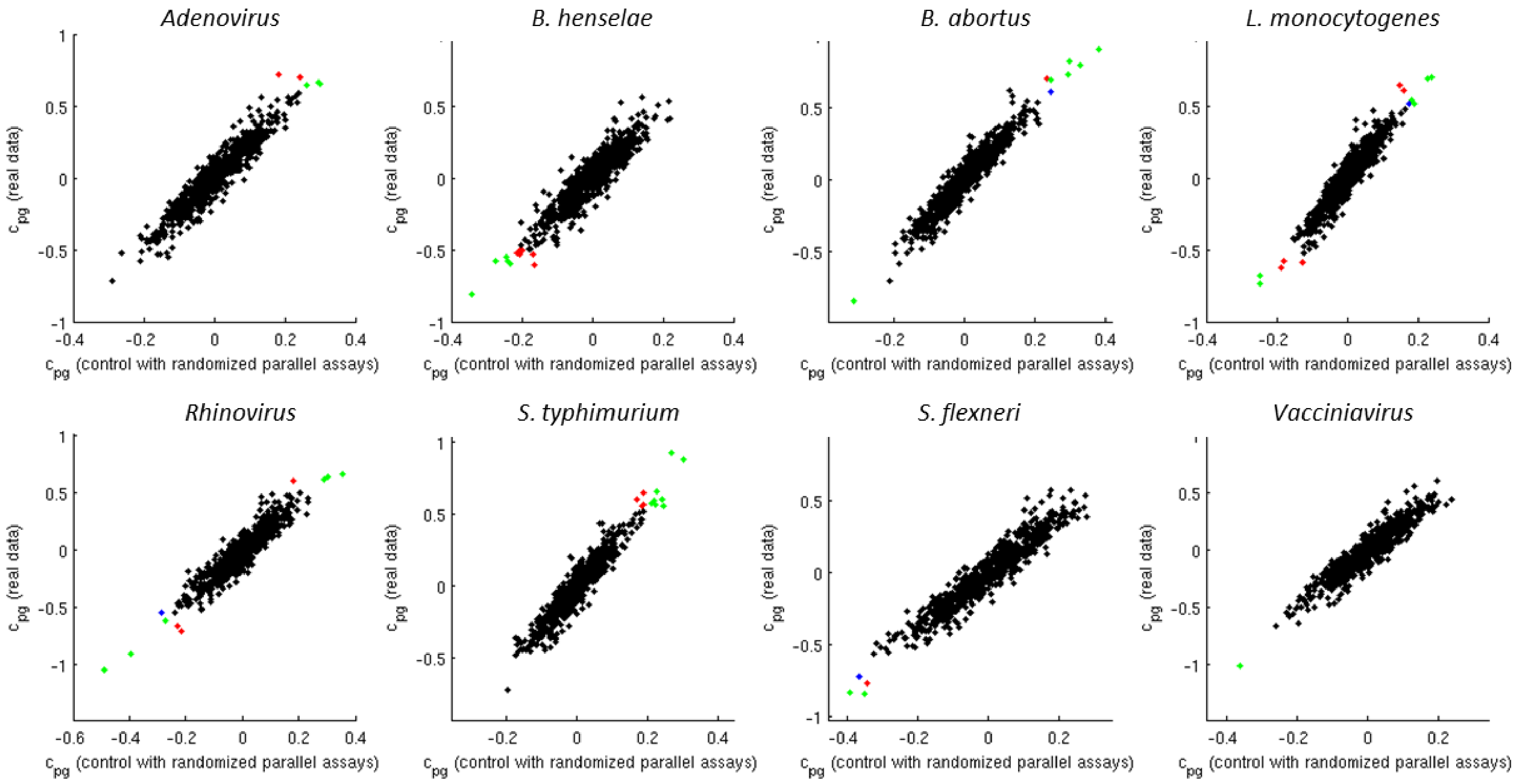

Supplementary Figure 7

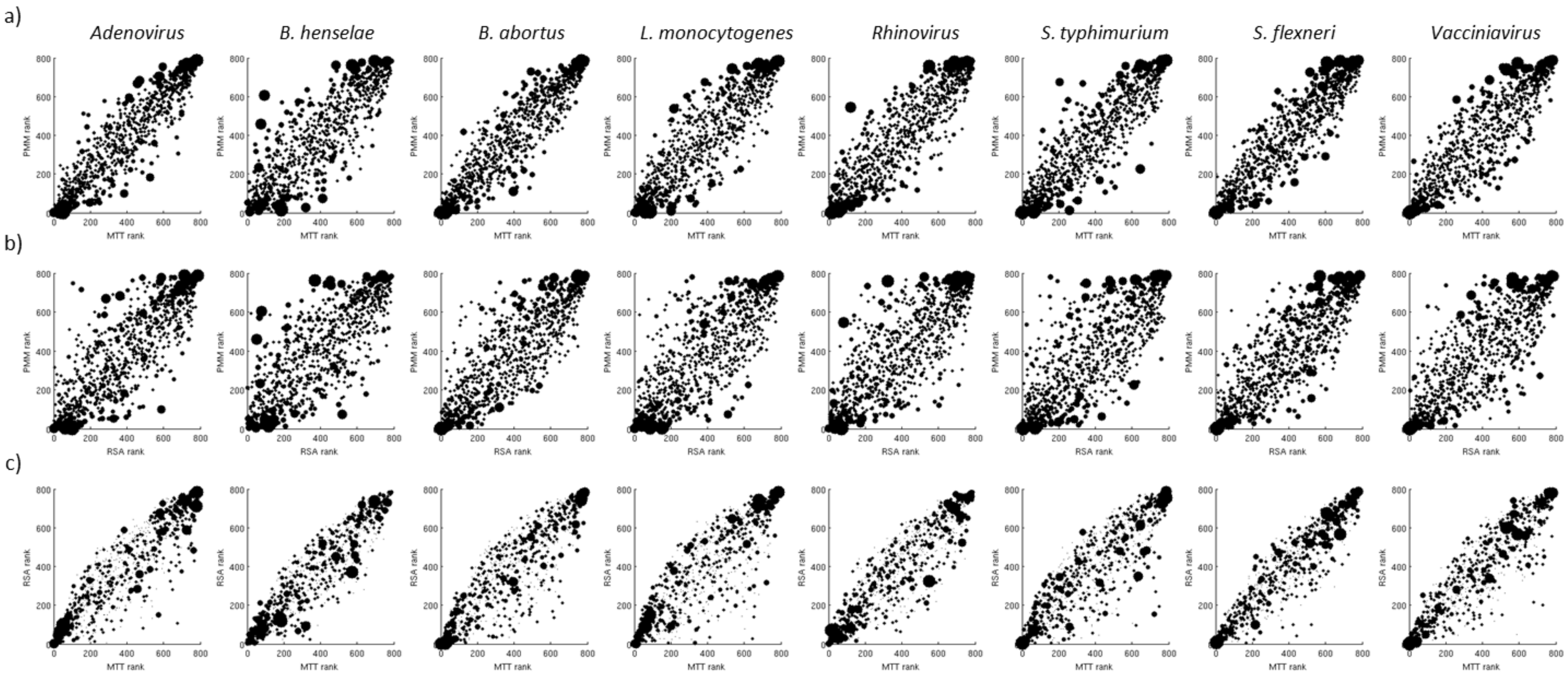

Supplementary Figure 8

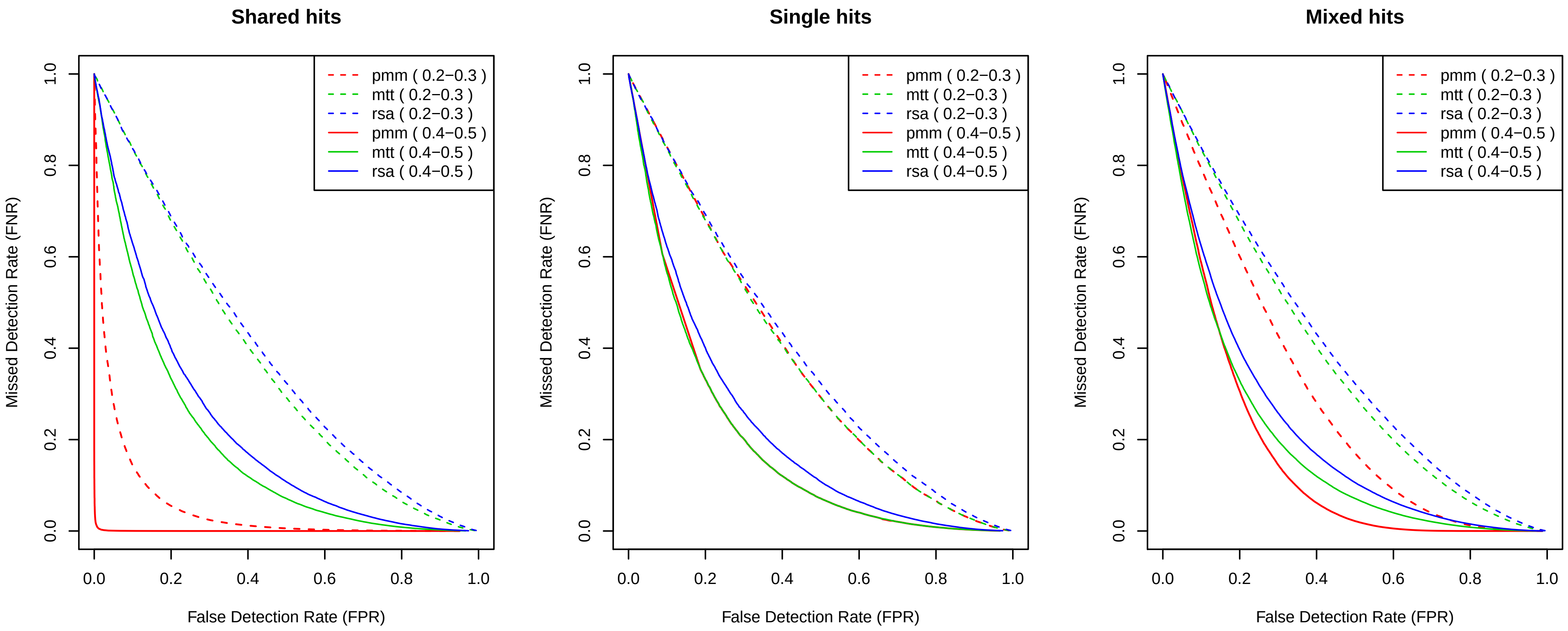

Supplementary Figure 9

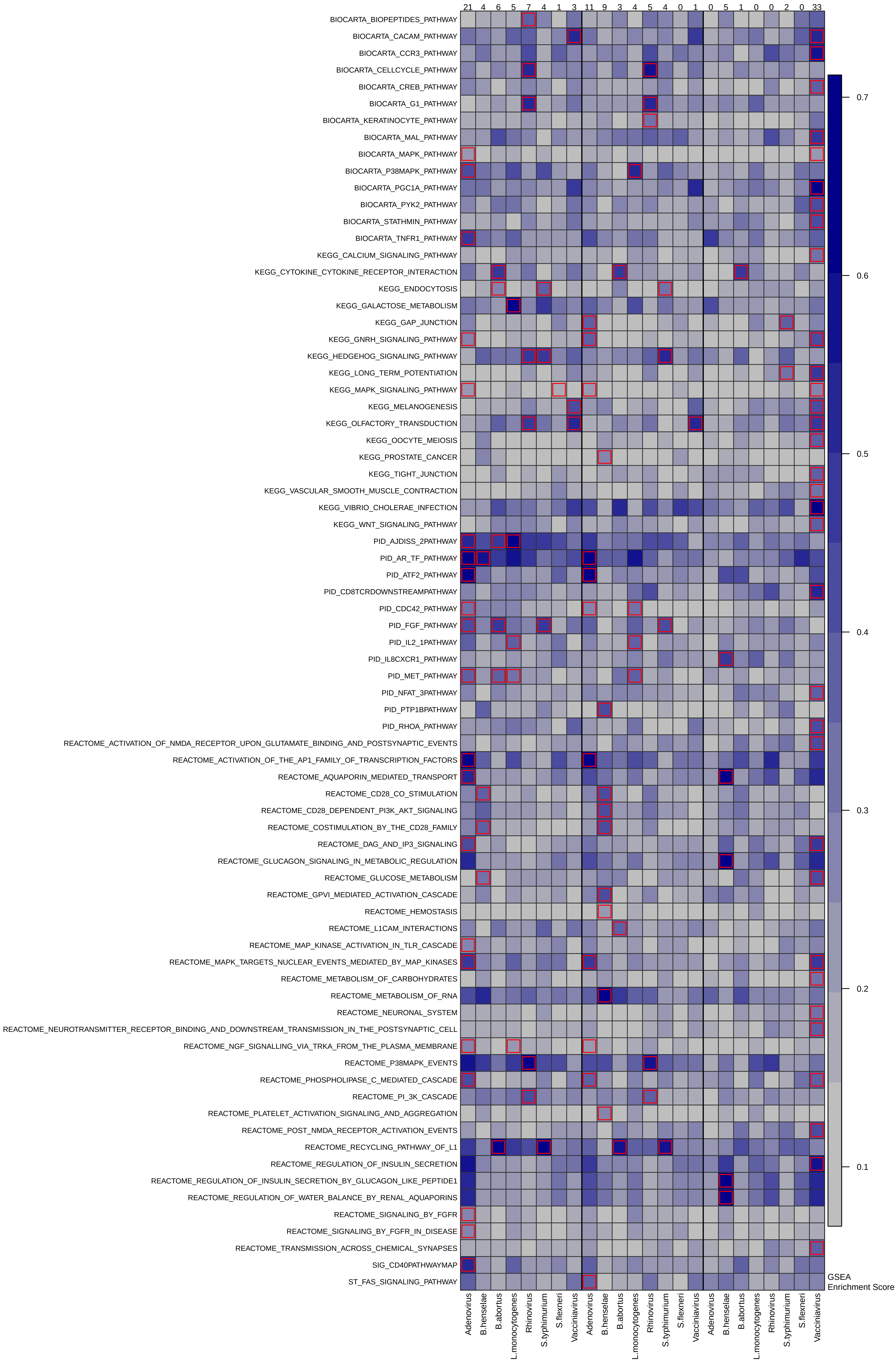

Supplementary Figure 10

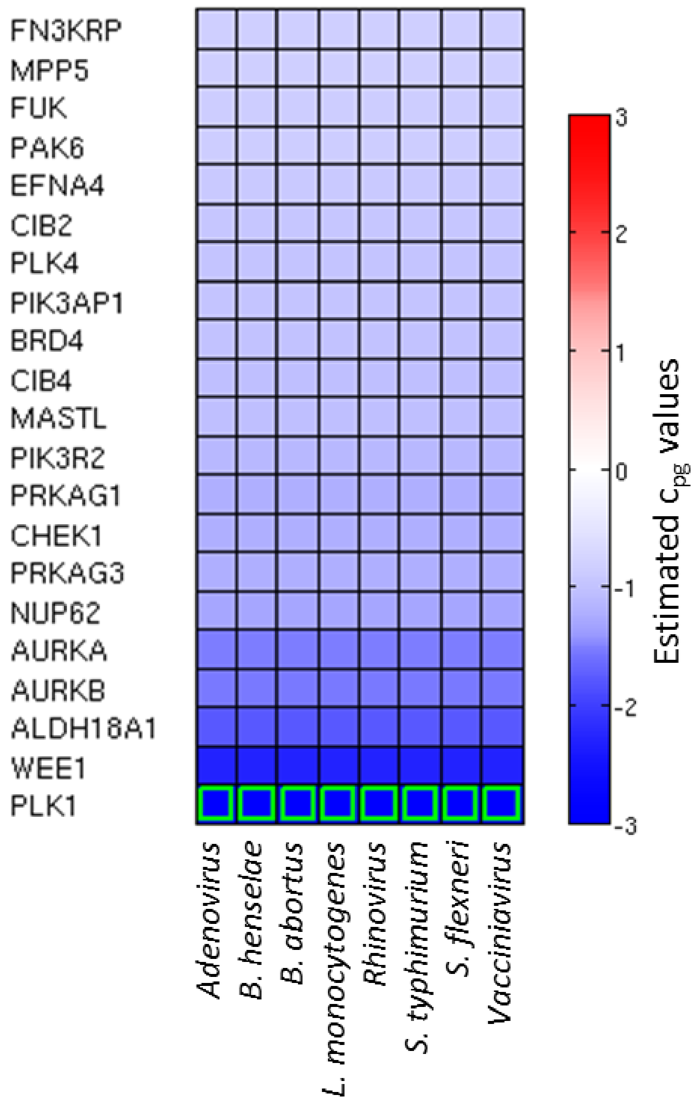

Supplementary Figure 11

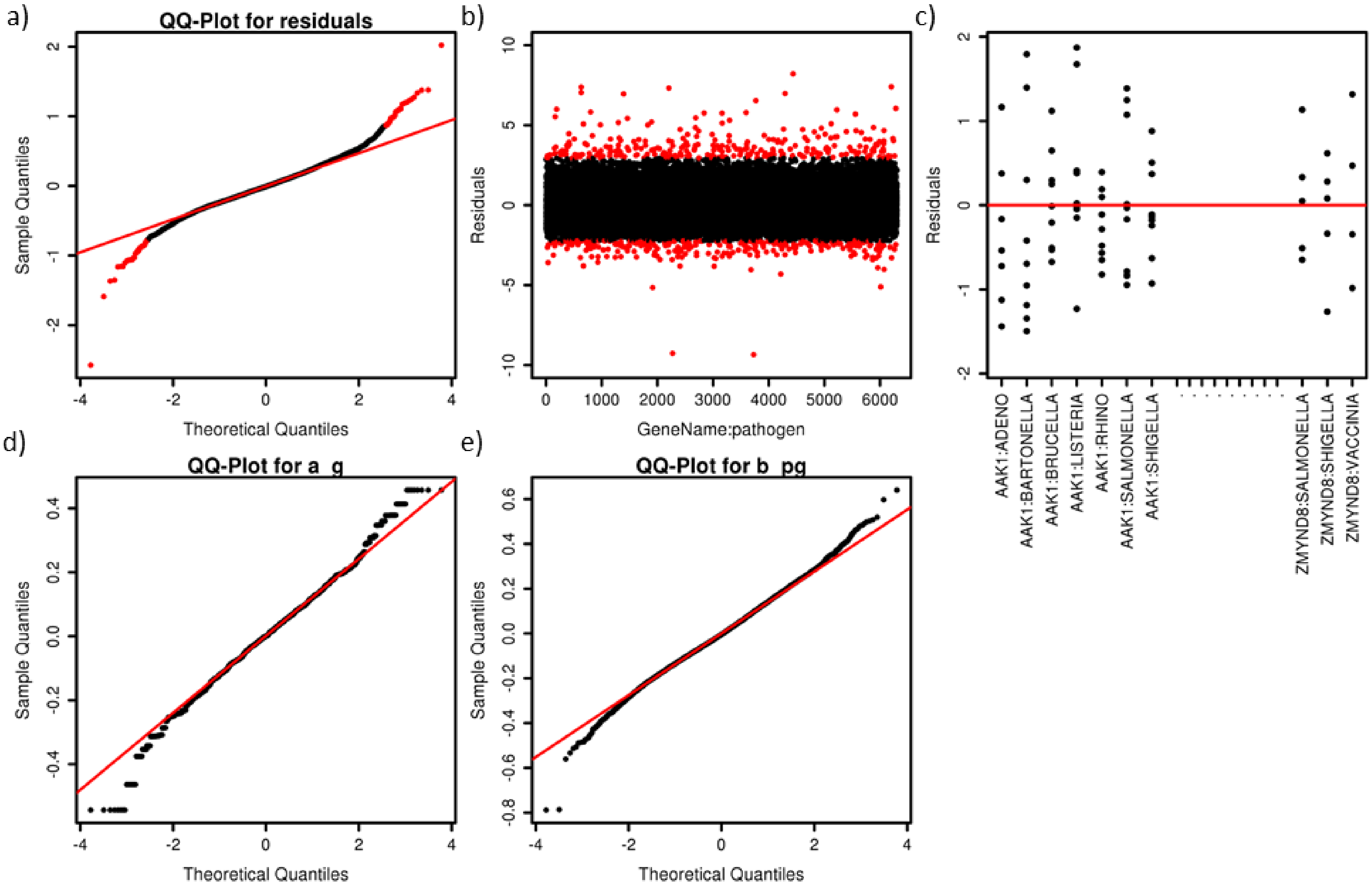

Supplement: Supplementary file 1 — Additional file 1: Supplementary Information. The additional data file 1 contains supporting information und further analysis results. (PDF 2 MB) [file 12864_2014_6988_MOESM1_ESM.pdf]
